# Baiting Insects with Pyrrolizidine Alkaloids (PAs): A Fieldwork-Oriented Review and Guide to PA-Pharmacophagy

**DOI:** 10.1007/s13744-023-01067-9

**Published:** 2023-09-01

**Authors:** Michael Boppré, Julio Monzón

**Affiliations:** 1 Forstzoologie und Entomologie, Albert-Ludwigs-Universität Freiburg, 79085 Freiburg i.Br, Germany; 2 Forstentomologie und Waldschutz, Albert-Ludwigs-Universität Freiburg, 79085 Freiburg i.Br, Germany

**Keywords:** Natural attractants, Lures, Plant secondary metabolites, Conservation, Biodiversity, Functional diversity, Arctiini, Danainae

## Abstract

Since 1890, many observations of danaine butterflies visiting dry plants of several families in the Old and New World tropics have been published. For 50 years, it has been recognised that Danainae, along with various other insects, seek out 1,2-dehydropyrrolizidine ester alkaloids (PAs) independently of and in addition to their nutritive requirements and utilise them to increase their chances for survival and biological fitness. This represents an unusual type of insect-plant relationship (“PA-pharmacophagy”), with remarkable peculiarities but also with gaps in knowledge, many of which can be filled by employing PA-baiting. We review and analyse the history of records on the attraction of adult insects to PAs and unveil the complex background information on PA-chemistry, PA-producing plants (“PA-plants”), and PA-sequestering insects (“PA-insects”) in order to come up with practical tips for successful baiting with PAs (“PA-baiting”). Studying PA-pharmacophagy integrates taxonomy, behaviour, and ecology with evolutionary biology, chemistry, and toxicology. With basic knowledge of PA-chemistry and bearing the general peculiarities of PA-plants and PA-insects in mind, PA-baiting can be conducted easily and successfully to address many questions on the multifaceted ecology of pyrrolizidine alkaloids. We aim to encourage field researchers in the tropics to employ PA-baiting as a valuable research method in this field of integrative biology.

**Supplementary Information:**

The online version contains supplementary material available at 10.1007/s13744-023-01067-9.

“Observe the butterflies, sombre black fellows (*Euploea*), flying in a crowd round a shrub with thick silvery-looking leaves. It is the *Tournefortia Argentifolia*, a tree that I see on almost every seashore ... A branch is broken, and the leaves are hanging dry and wilted. The butterflies settle on the dead leaves in swarms, almost pushing and jostling one another to get a good place. Notice that it is the withered leaves and flowers that they prefer, and seem to become half-stupid in their eagerness to extract the peculiar sweetness, or whatever it is, that the leaves contain.” (Woodford 1890:94)

## Introduction

Besides the *Euploea* butterflies (Lepidoptera: Nymphalidae: Danaini) observed by Woodford (1890), various other adult insects visit certain dry plants. Edgar et al. (1973), Edgar (1975), and Schneider et al. (1975) found that the attractive plants are characterised by the presence of 1,2-dehydropyrrolizidine ester alkaloids (PAs). Insects that take up these pro-toxic plant secondary metabolites (PSMs) store them for defence against antagonists (e.g. Edgar et al. 1979; Brown 1984; Cardoso 1997; Trigo 2000, 2008, 2011; Nishida 2002; Silva and Trigo 2002). For many male Lepidoptera, PAs are also essential precursors to the biosynthesis of courtship pheromones that are decisive for female mate choice (e.g. Meinwald et al. 1969; Edgar et al. 1971, 1973, 1976; Brown 1985; Schulz 1998, 2009; Schulz et al. 2004; Conner 2009). The syndrome of PA-pharmacophagy—the search for and uptake of PAs by insects not for nourishment but to potentially increase chances of survival and biological fitness—requires further elucidation. Using PA-plants to bait PA-insects can expand basic knowledge but is rarely employed. Accordingly, many interesting aspects related to taxonomy, ecology, faunistics, zoogeography, evolutionary biology, and physiology are yet to be well studied.

Here, we provide an overview of reports on adult insects visiting withered PA-plants, summarise current knowledge, identify research deficiencies, discuss principles and variables that influence PA-baiting, and offer practical advice to field entomologists on how to successfully employ PA-baiting; much personal experience, yet unpublished, is included. While omitting many details on functional aspects, the focus is on application of current knowledge to fieldwork. This paper does not aim to comprehensively address the ecology of PAs; rather, its objective is to raise awareness and inspire amateur and professional entomologists in the tropics to contribute to closing knowledge gaps by employing PA-baiting. We quote only a fraction of the literature available in the wider context of insect-PA relationships but reviews, including Boppré (1978, 1986, 1990, 2011), Brown (1985), Hartmann and Witte (1995), Hartmann (1999, 2009), Hartmann and Ober (2000, 2008), Conner and Weller (2004), Honda (2008), and Trigo (2008, 2011), provide many references. Some details and additional aspects are discussed in Supplementary Information (SI).

The essential roles of PAs for certain insects contradict the risks that PAs pose to livestock and human health through feed and food, culinary herbs, herbal teas, spices, and honeybee products (IPCS 1988; EFSA 2011, 2017; Wiedenfeld and Edgar 2011; Boppré 2011; JECFA 2020). PAs are also discussed as promising prototypes for the development of new drugs (Wei et al. 2021). Although the health issues place entomological studies on PAs in a human context and thus give them additional relevance, they are not addressed here. PA-intoxication, which in humans is typically delayed and quite insidious, explains why the literature on PAs grows steadily. Publications deal on hepatotoxic, genotoxic, carcinogenic, and teratogenic effects, analyses of herbal medicines, food and feed, metabolism in vertebrates, refining extraction and detection procedures; they indirectly touch the topic under discussion here. Recent reviews on the occurrence of PAs and/or their chemical classification include Mroczek and Glowniak (2002), Ma et al. (2018), Moreira et al. (2018), and Tamariz et al. (2018).

## Reports on insects that visit sources of pyrrolizidine alkaloids

Woodford’s (1890) initial observation of adult Lepidoptera exhibiting a relationship with withered parts of “*Tournefortia Argentea*” (now *Heliotropium foertherianum*; Boraginaceae) was made in the Old World tropics. It was followed by similar anecdotal reports also from the New World tropics involving not only numerous other plants but additional insect taxa, too. Publications on adult insects found visiting PA-plants are listed and commented in Tables 1 and S1.

**Table 1:** Table 1 Chronology of original reports with principal news on insects attracted to plants containing pyrrolizidine alkaloids (PAs) with references and comments. ^1^After Holt et al. (2013). Column type: **I** incidental observations, **E** experimental baiting. **AGA** Aganainae, **APO** Apocynaceae, **ARC** Arctiini, **AST** Asteraceae, **BOR** Boraginaceae, **DAN** Danaini, **DIP** Diptera, **FAB** Fabaceae, **ITH** Ithomiini, **ORC** Orchidaceae, **ORT** Orthoptera. Table S1 provides supplementing reports; to the best of our knowledge, Tables 1 and S1 combined comprise all published reports on field records. For details on insects and plants, see Tables S2 and S3, respectively

Reference	Zoogeographic realm^1^	Country	Type	Plant	Insect	Major news, quotes, comments
Woodford (1890)	Oceanian	Solomon Islands	I	BOR	DAN	Crowd of *Euploea* butterflies at dry leaves of a broken branch of *Heliotropium foertherianum* (as *Tournefortia argentifolia*) First report ever on PA-pharmacophagy
Jörgensen (1913)	Neotropical	Argentinia	I	AST	ARC	Wondering about “teeming clumps of hundreds of individuals” of ARC (as Syntomidae) at flowers but also on green plant parts that had been wounded by bees and wasps as well as at dry stems and leaves of *Senecio brasiliensis* First report of day-flying moths visiting a PA-plant; first report from the Neotropics; first report on attraction to an Asteraceae. See SI 7 for comment
Subrahmaniam (1920)	Oriental	India	I	FAB	DAN	“... a large swarm of *Danais* [*Tirumala*] *limniace*” at *Crotalaria striata* “... very busy scratching up the surface of the pods with the claws of their middle pair of legs in a steady and persistent manner, the tip of the uncoiled proboscis following the scratched portions ... sucking up the juice oozing out ...” First report on attraction to a FAB
Hopkins & Buxton (1926)	Oceanian	Samoa, Tonga	I	BOR	DAN	*Euploea* and *Danaus* (as *Danais*) “the males of which frequent *H. foertherianum* (as *T. argentea*) trees ... the flowers are apparently unattractive, but the butterflies are to be seen in swarms of many hundreds on withered fruit-clusters and broken branches. ... The explanation of this habit is not known”
Zerny (1931)	Neotropical	Brazil	E	BOR	ARC	Used, based on personal information from Hagmann (1938), dry *H. indicum* as bait and collected 78 species in 37 genera of Ctenuchina and Euchromiina. No mention of Pericopina and Phaegopterina, likely because of specific interest in Syntomidae First report on baiting with a PA-plant; first report on moths attracted at night; first sp. nov. found at PA-bait
Lever (1936)	Oceanian	Solomon Islands	I	BOR	DAN ARC	*Euploea* and *Euchromia oenone* at dry *H. foertherianum* (as *T. argentea*). “I found a few withered flowers and their adjacent leaves, which were brown and damp. These shoots I collected and pulled off, also some branches with fresh green leaves, and placed the flowers and withered leaves in one heap and the living branches in a heap on either side. In a few minutes the central pile was visited by up to half a dozen male Euploeas, ...” “Incidentally the planter with whom I was staying referred to the central heap as the “bait,” an apt name.” First report of a diurnal ARC visiting a PA-plant in the Old World
Hagmann (1938)	Neotropical	Brazil	I, E	BOR	ARC	Observed ARC (as Syntomidae) by accident at *H. indicum* and used the plant subsequently as bait in areas where it was not growing; list of species collected at light and bait provided but not differenciated. See SI 7 for comment
Beebe (1955)	Neotropical	Trinidad	E	BOR	DAN ARC	Used *Heliotropium* as bait, following Hagmann (1938). “All this was revolutionized the following season by the use of the common weed, *Heliotropium indicum* Linnaeus, which proved to be a remarkably efficient and selective attractant.” “Until we began to use this method of collecting we had never come across a specimen of *Sphecosoma trinitatis* Rothschild, ... In the course of five months we took or observed one hundred and forty-seven of these wasp-mimicking moths on fedegoso, and these resolved into three distinct species, two of which had not heretofore been recorded from Trinidad.” First photographs of Lepidoptera at a PA-plant
Morrell (1955)	Oriental	Singapore, Malaysia	E	BOR	DAN AGA ARC	Tested *H. indicum* as bait on behalf of W. Beebe and found several *Euploea, Danaus*, *Ideopsis gaura*, and a few moths First report on nocturnal moths visiting a PA-plant in the Old World; first report of an AGA visitingt a PA-plant
Masters (1968)	Neotropical	Venezuela	E	BOR	ITH ARC	Account on baiting ITH with *H. indicum,* “a secret of a few professional collectors”; was informed in 1965 by an experienced collector, Albert Gadou, and “... collected perhaps 300 individuals during one productive day ...”; *Sphecosoma* sp. which were "otherwise quite scarce in the area" also attracted First report on ITH visiting a PA-plant
Sevastopulo (1969)	Afrotropical	Kenya	E	BOR	DAN	Tested, stimulated by literature, dry *H. indicum* as bait in East Africa and found *Amauris niavius* to be attracted First report on butterflies visiting a PA-plant in Africa
Negishi (1971)	Neotropical	Venezuela	E	BOR	ITH ARC	Conducted an inventory of ITH with *H. indicum* baits and collected >2,000 specimens of >40 species; mentions ARC (as Synthomidae [sic; recte Syntomidae]) without details
**Edgar et al.** (1973)**, Edgar** (1975)**, and Schneider et al.** (1975)**: Recognition that attractiveness is due to the presence of 1,2-dehydropyrrolizidine ester alkaloids (PAs)**						
Wagner (1973)	Nearctic	USA: Michigan	I	ORC	DAN	“Invasion” of *Danaus plexippus* in an orchid greenhouse with some flowering *Epidendrum paniculatum* plants at Ann Arbor Botanical Garden seemingly paralleling attraction to *Heliotropium* See SI 5 for comment First report on attraction of a DAN to an ORC
Atkins (1974a, b)	Australasian	Australia	I, E	APO BOR FAB	DAN	Species of *Danaus*, *Tirumala*, and *Euploea* at *H. amplexicaule*, *C. spectabilis*, *C. lanceolata*, *Parsonsia straminea* and *P. eucalyptophylla* First report on butterflies visiting PA-plants in Australia; first report on attraction to an APO
Pliske (1975a, b)	Nearctic, Neotropical	USA: Florida, Panama, Ecuador, Trinidad, Venezuela	E	AST BOR	DAN ITH ARC DIP	Baiting in several countries with several plants (*Senecio glabellus, Eupatorium capillifolium, E. coelestinum, C. spectabilis, H. curassavicum, H. angiospermum, Tournefortia gnaphalodes*) and finding 49 spp. of ITH, 2 DAN and 179 ARC, of some hundreds of individuals of many only a few *Chromolaena xestolepis* (as *Eupatorium*) is almost exclusively pollinated by ITH and ARC but dead flowers and leaves are never attractive. See SI 5 for PAs in nectar First study comparing bait plants and habitats; first report of DIP visiting PA-plants. See SI 7 for comment
Goss (1979)	Nearctic	USA: Florida	E	AST BOR FAB	ARC	Studied ARC at roots of several PA-plants, in particular *E. capillifolium.* Evaluation of age, mating status, sex bias and comparison of attraction to light *vs* PA-sources First cage tests on attraction to PA-plants; first study on physiological aspects. See SI 7 for comment
Boppré (1981)	Afrotropical	Kenya	E	BOR	ARC DAN	Species of *Danaus*, *Amauris, Tirumala, Euchromia*, *Nyctemera,* and *Amerila* (as *Rhodogastria*) at *Heliotropium* baits. First report of ARC visiting PA-sources in Africa
Boppré & Scherer (1981)	Afrotropical	Kenya	E	BOR	COL	*Gabonia gabriela* sp. nov. collected at PA-baits. First report of a COL visiting a PA-plant
**Boppré** (1984)**: Coining of the term "pharmacophagy"**						
Boppré et al. (1984)	Afrotropical	South Africa	E	pPA	ORT	*Zonocerus elegans* attracted to dishes containing pure PAs First report of an ORT visiting PA-sources; first report of insects visiting dishing containing pure PAs
DeVries (1987)	Panamanian	Costa Rica	I, E	AST BOR	ITH	“In Costa Rica the major plant sources highly attractive to ithomiine males are *Heliotropium*, *Tournefourtia* [sic; recte *Tournefortia*], *Myosotis* (Boraginaceae), *Eupatorium*, *Neomirandia* [sic; recte *Neomirandea* ?], and *Senecio* (Asteraceae). It is good field practice always to carry some dead Boraginaceae. By hanging bits of these plants along a trap line it is possible, over the course of several days, to get a very good estimate of which ithomiine species occur there. The males of most species and both sexes of other species avidly visit these baits within an hour of placing them in the forest”
Sourakov & Emmel (2001)	Oceanian	Solomon Islands	I	BOR	DAN ARC AGA	*Euploea* spp.*, Euchromia collaris,* and *Asota* spp. at *H. foertherianum* (as *Tournefortia*). “... hundreds of *Euploea* danaids came to the dying leaves, branches and decomposing plant matter of Tree Heliotrope ...”. “Local people of New Georgia ... call the heliotrope a “butterfly tree”. “... hundreds of brightly colored arctiids ...”. “even old charred coals and half-burned branches of this coastal plant were loaded with feeding butterflies” First report of moths visiting a PA-plant in Oceanian
Brehm et al. (2007)	Neotropical	Ecuador	I, E	APO	ITH ARC	Found ITH at withered flowers of *Prestonia amabilis*, used roots as bait and attracted ITH and ARC First report on attraction to an APO in the New World
Krauska (2009)	Nearctic	USA: Missouri	I	AST	DAN	*Danaus plexippus* attracted to withering roots of introduced *Gymnocoronis spilanthoides*, subsequently found to contain PAs. First report on attraction to an aquatic PA-plant. No mention of PAs
Grados et al. (2015)	Neotropical	Peru	E	BOR	ARC	Compared collecting ARC with light *vs Heliotropium* bait; within 16 days 97 species were collected at the bait, 63 at night and 39 during day, 48 exclusively at the bait. List of species provided but not differenciated. No mention of PAs
Colegate et al. (2016)	Afrotropical	Kenya	I, E	APO	DAN COL	DAN and *Gabonia* beetles attracted to roots of *Alafia* cf. *caudata*; *Aganosma marginata* and roots of *Echites panduratus* (as *Fernaldia*) attractive for PA-insects in the lab First report on attraction to an APO in Africa and presence of PAs in African APO
Tea et al. (2021)	Oriental	Sulawesi	I	APO	DAN	Several species of DAN at PA-plants but also scratching and sucking at *Idea* caterpillars feeding on *Parsonsia*. Coined “kleptopharmacophagy” and other terms. See SI 1 for comment
Vincent et al. (pers. comm. 2022)	Neotropical	French Guyana	E	AST	ARC	22 species of ARC collected within four days only during daytime at roots of *Chromolaena odorata* First report of baiting ARC with a *Chromolaena* sp

**Fig. 1:**
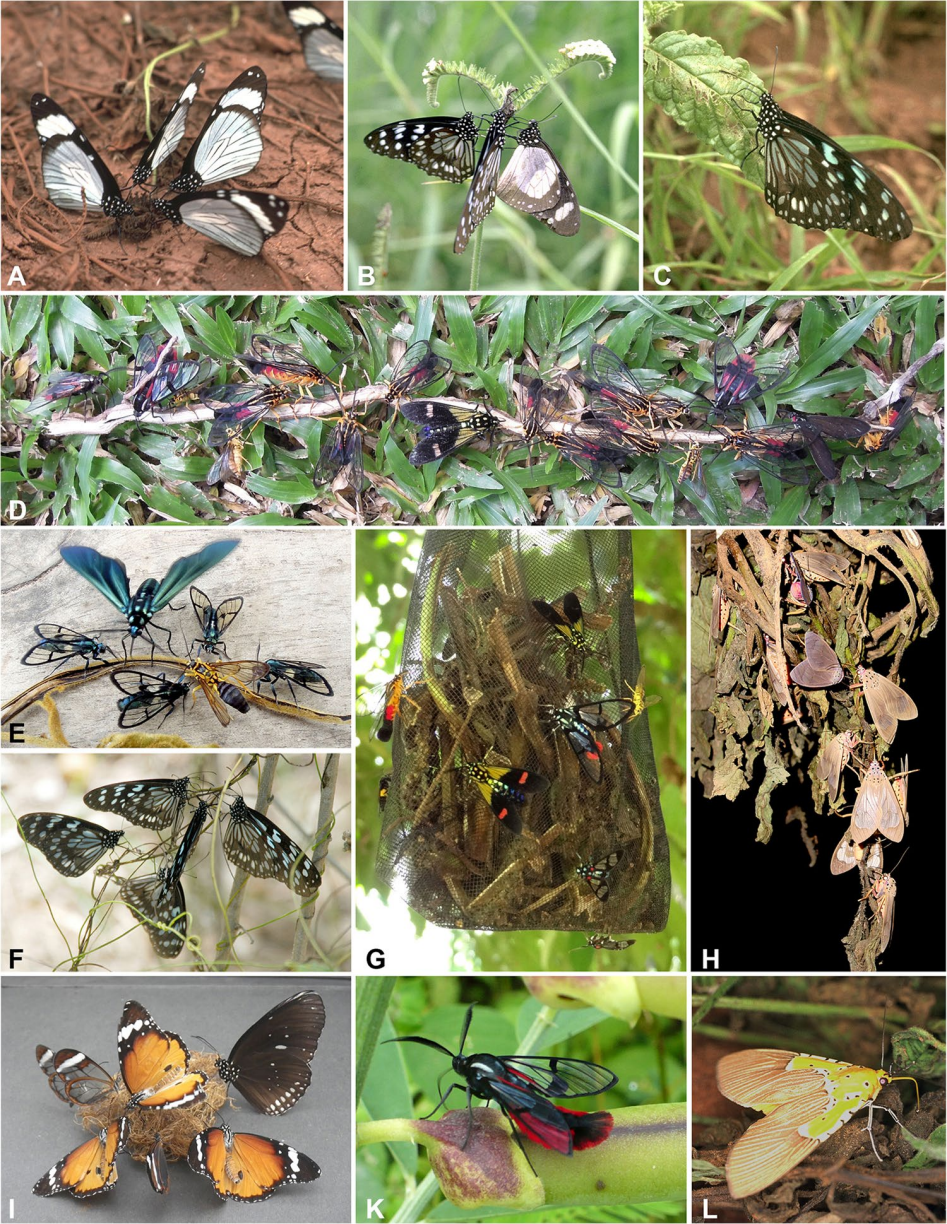
Insects that consume pyrrolizidine alkaloids from natural sources (A–C, F, K) and from PA-containing plant material used as baits (D, E, G–I, L). (**A**) *Amauris niavius* (Linnaeus, 1758) (Danaini) on rotting parts of *Heliotropium* sp. (Boraginaceae). (**B**) *Tirumala petiverana* (Doubleday, [1847]) and *A. ochlea* (Boisduval, 1847) (Danaini) on a wound of an inflorescence of *Heliotropium*. (**C**) *T. petiverana* scratching at holes made by flea beetles on *Heliotropium* leaf. (**D**, **E**) Diurnal Arctiini on a twig of an undetermined plant apparently containing PAs. (**F**) *T. petiverana* on dry *Cassytha* sp. (Lauraceae) that is parasitising a PA-containing *Crotalaria scasellatii* (Fabaceae). (**G**) Day-active Arctiini. (**H**) *Amerila* spp. on *Heliotropium*. (**I**) *Greta oto* (Hewitson, 1854) (Ithomiini, South American), *Euploea core* (Cramer, 1780) (Danaini, Asian), and *Danaus chrysippus* (Linnaeus, 1758) (Danaini, African) gathering PAs from dry roots of *Gymnocoronis spilanthoides* (Asteraceae, South American) in captivity. (**K**) *Dinia aeagrus* (Cramer, [1779]) (Arctiini) on a seedpod of *Crotalaria* sp. (**L**) *Asota speciosa* (Drury, 1773)(Aganainae) on *Heliotropium* sp. Pictures were taken in Kenya (A–C, F, L), Peru (D, E, G), Ghana (H), our greenhouse in Germany (I), and Costa Rica (K)

Incidental observations (type I in Tables 1 and S1) seem rare or are infrequently reported; almost all mention groups of butterflies and relatively few express curiosity. With no knowledge about earlier reports, and already decades before it was understood why insects visit dry plants, in Brazil, Zerny (1931) and Hagmann (1938) experimentally set up uprooted *Heliotropium indicum* as baits, checking them during the day and at night. They attracted many species of arctiine moths, some of which were otherwise poorly collectable or even unknown. Since then, however, baiting with PA-plants has been conducted only quite infrequently at a few spots and for short periods in Latin America, and even more rarely in Asia, Australia, and Africa (type E in Tables 1 and S1). Pliske (1975a) was aware of PAs and not only baited insects with different PA-plants in several habitats but reported many taxa and basic details (see SI 6). Although sporadic, these studies show that using PA-plants as lures is useful for recording and collecting a diverse range of insects (Figs. 1 and 4 and Table S2). So far, species of four orders (mainly Lepidoptera: Nymphalidae: Danainae: Ithomiini and Danaini; Erebidae: Arctiinae and Aganainae; Orthoptera: Pyrgomorphidae; Diptera: Chloropidae; Coleoptera: Chrysomelidae) have been found to be attracted to a variety of plant species belonging to the Apocynaceae, Asteraceae, Boraginaceae, and Fabaceae (Tables 1, S1, and S3) when these plants are withered or injured; insect attraction occurs both during the day and at night.

**Fig. 2:**
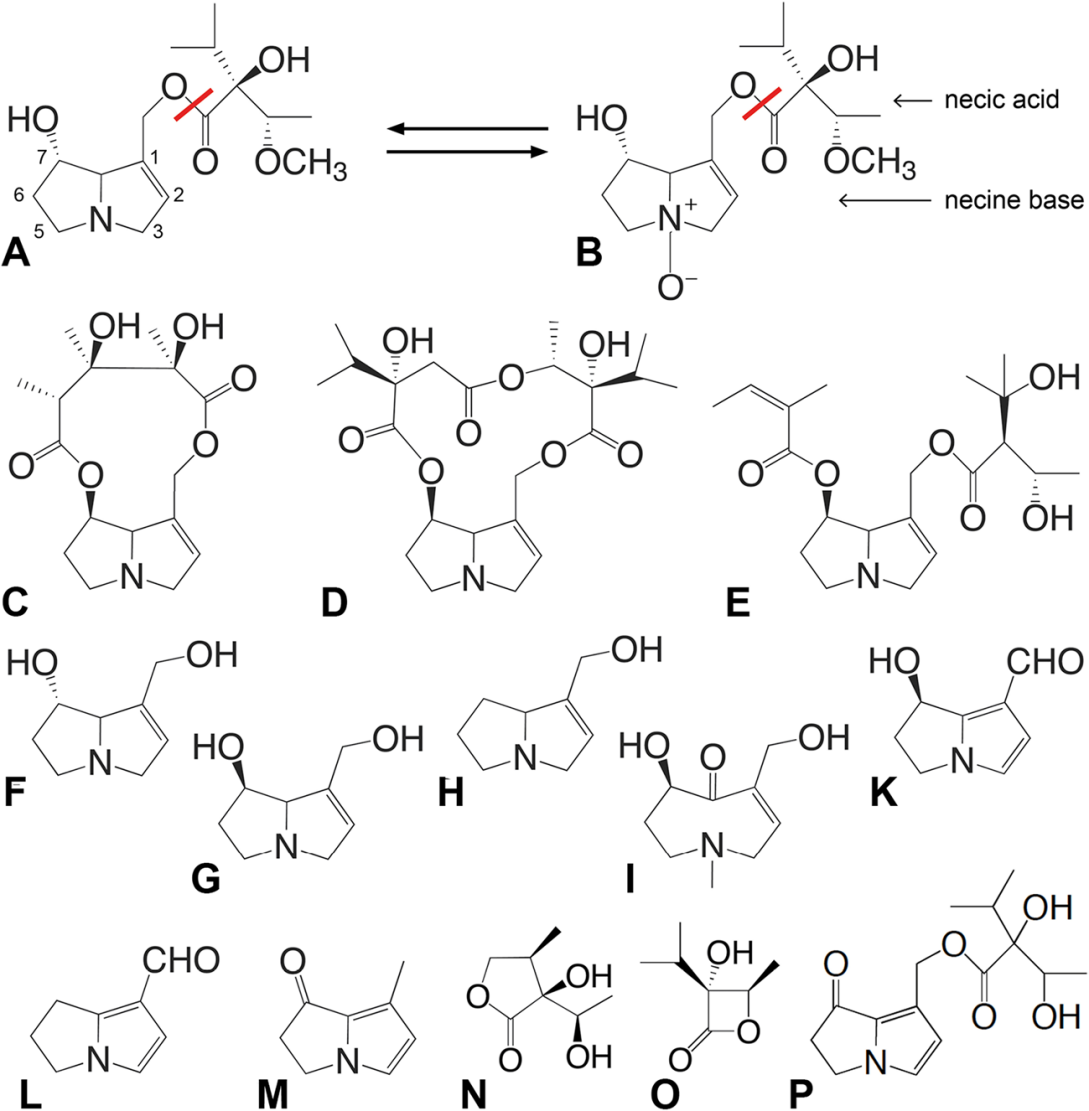
Examples of molecular structures of plant 1,2-dehydropyrrolizidine ester alkaloids (**A**–**E**), necine bases (**F**–**I**), volatile insect-synthesised PA-derivatives ((**K**–**M**): dihydropyrrolizines; (**N**, **O**): lactones) that are used as male courtship pheromones, and a plant dienone compound (**P**). (**A**) Heliotrine (monoester). (**B**) Heliotrine *N*-oxide ((A) and (B) interconvert; red line: site of hydrolysis). (**C**) Monocrotaline (cyclic diester). (**D**) Parsonsine. (**E**) Echimidine (diester). (**F**) Heliotridine. (**G**) Retronecine. (**H**) Supinidine. (**I**) Otonecine. (**K**) Hydroxydanaidal. (**L**) Danaidal. (**M**) Danaidone. (**N**, **O**) Lactones. (**P**) Parsonine. See text and SI 3

**Fig. 3:**
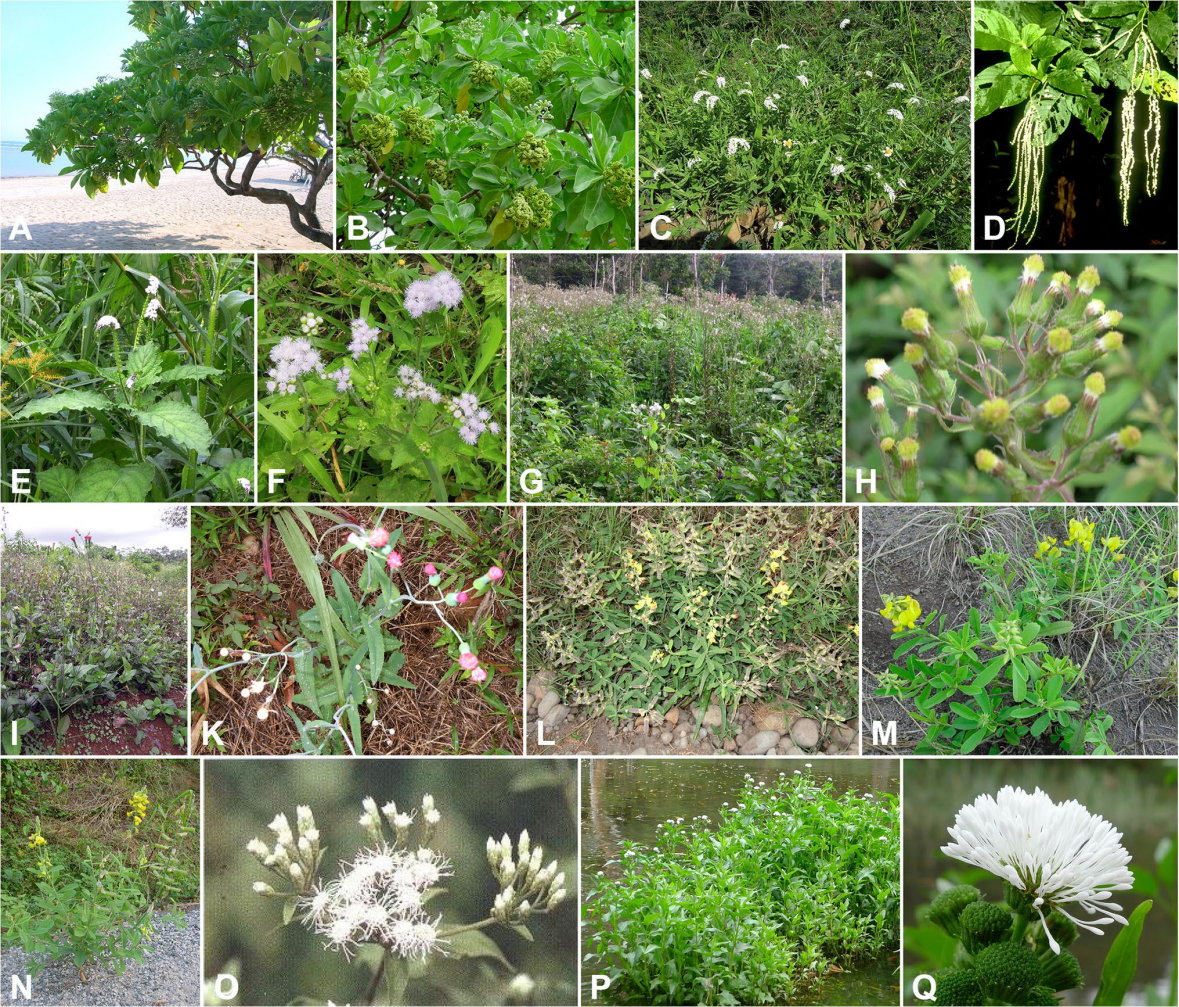
Examples of plants that are attractive to PA-pharmacophagous insects when injured, wilted, or dry (cf. Table S3). (**A**, **B**) *Heliotropium foertherianum*. (**C**, **E**) *H. indicum*. (**D**) *Tournefortia* sp. (**F**) *Ageratum conyzoides*. (**G**, **H**) *Erechtites hieraciifolius.* (**I**, **K**) *Emilia* sp. (**L**–**N**) *Crotalaria* spp. (**O**) *Chromolaena odorata*. (**P**, **Q**) *Gymnocornis spilanthoides*

**Fig. 4:**
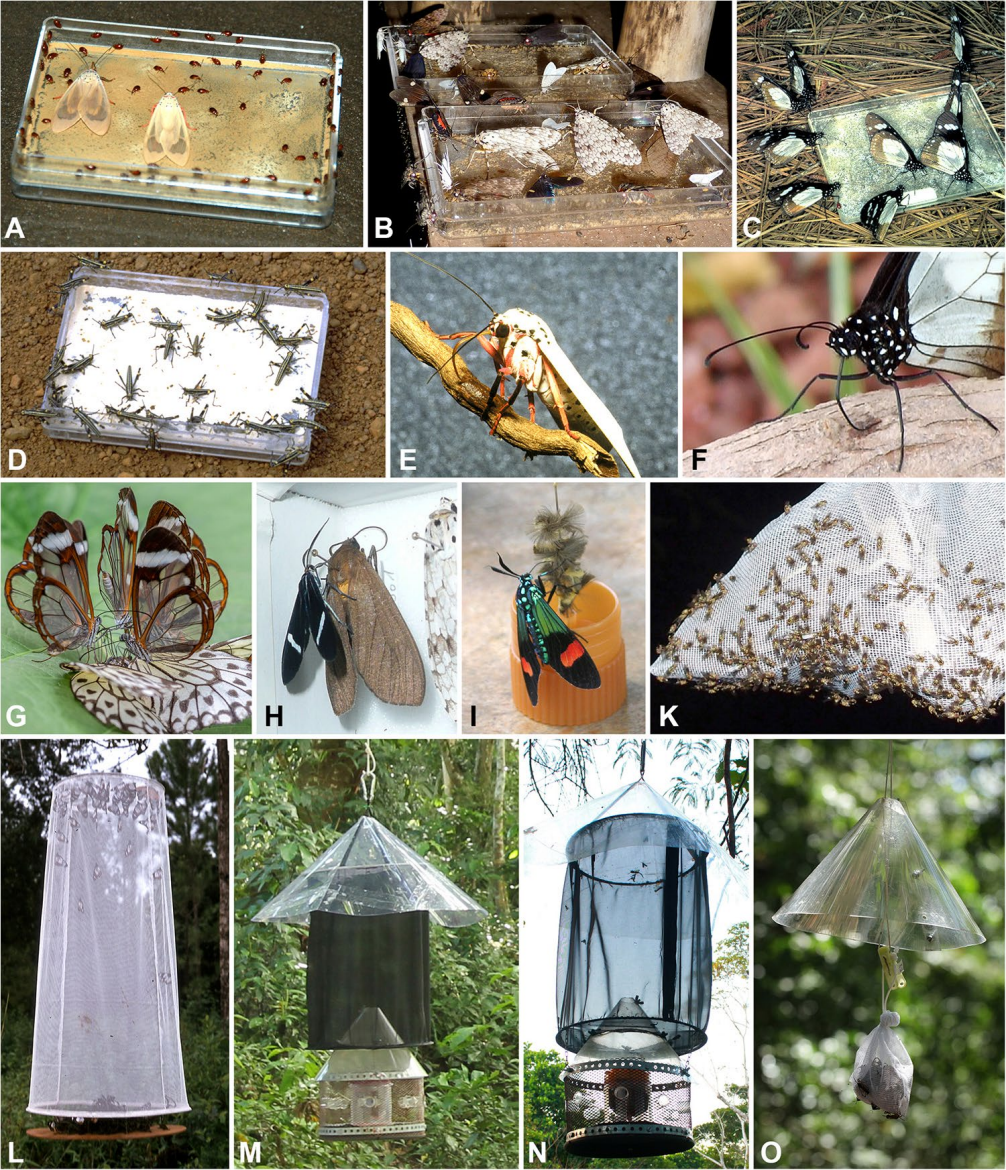
(**A**–**D**) Insects gathering pyrrolizidine alkaloids (PAs). (**A**) *Gabonia* spp. and *Amerila* sp. (**B**) Various Arctiini. (**C**) *Amauris ochlea*. (**D**) Nymphs of *Zonocerus variegatus* at dishes containing pure PAs. (**E**, **F**) *Amerila* sp. (**E**) and *A. ochlea* (**F**) applying fluid on dry plant matter to dissolve PAs. (**G**) *Greta oto* (Ithomiini) gathering PAs from a carcass of *Idea leuconoe* (Erichson, 1834) (Danaini). (**H**) *Aclytia* sp. sucking on a pinned *Elysius* sp. (**I**) *Belemnia inaurata* (Sulzer, 1776) (Arctiini) on hydroxydanaidal-containing coremata separated from *Creatonotos transiens* (Walker, 1855) (Arctiini). (**K**) Chloropidae on a bag with dry *Heliotropium indicum*. (**L**) A typical butterfly trap with *Heliotropium* and Danaini. (**M**, **N**) Prototypes of a new bait trap for moths. (**O**) A bag with a PA-bait hooded for protection from rain

While it can be concluded that PA-baiting merits frequent application, surprisingly, it has not often been employed in many habitats nor for extended periods (Tables 1 and S1). Limited availability of PA-plants may restrict the preparation of baits, or perhaps their temporary attractiveness is frustrating. Ignorance of background facts or of the significance of the syndrome, and/or unsuccessful attempts could have made PA-baiting seemingly unappealing. Indeed, on a ‘good day’, dozens of specimens can be attracted to a PA-bait within a short time, but on another day, even in the same habitat, no or very few insects visit; one must be impervious to disappointment and ready for excitement at the same time.

The success of PA-baiting is determined by several intertwined factors that are yet to be comprehensively communicated. Much has been published but is hidden in widely unavailable journals (e.g. Häuser and Boppré 1997a; Boppré 1997, 1999) or in articles which focus on chemical, medicinal, or botanical matters; many papers address the context superficially or include misconceptions and false statements due to ignorance or insufficient experience. For example, Morrell (1955) stated that “the bait should not be protected from the rain”—but rain washes out PA *N*-oxides (see below). Some authors mention PA-pharmacophagy without including PA-baits in their studies (e.g. Marinoni and Dutra 1996; Hilt and Fiedler 2006; Zenker et al. 2015); others employed baiting with *Heliotropium* successfully without contextualising it with PAs (Hernández-Baz and Grados 2004) or without even mentioning PAs at all (e.g. Cock 2003; Grados et al. 2015; Hernández-Baz et al. 2016). Tea et al. (2021) observed danaines gathering PAs from caterpillars, coined a redundant term (see SI 1), and generated excessive discussions in the media. Unfortunately, authors who made casual observations did not continue to observe the attractive plant, such as checking it at night or testing it in another habitat, even when they knew about PA-pharmacophagy (e.g. Sourakov and Emmel 2001).

In summary, the literature provides a patchy and partly confusing picture of PA-baiting and PA-pharmacophagy, which obscures this exciting biological syndrome in an unscientific way.

## Insects that sequester pyrrolizidine alkaloids

### Adult insects that visit withered or injured PA-plants

Butterflies with uncoiled proboscises on dead or injured plant tissue (Figs. 1 and 4) provide a counterintuitive sight because one would expect to see them gathering nectar from flowers. Close inspection reveals that the butterflies, using their proboscises, regurgitate a liquid (gut content and/or saliva) onto the dry plant tissue (Fig. 4E and F) and re-imbibe it, enriched with dissolved PAs.

Although all plants that are attractive to adult insects when withered or injured contain PAs, this does not necessarily mean that PAs and not any other compounds (e.g. those responsible for the strong smell of some dry PA-plants to humans) elicit the attraction. However, proofs that these insects’ response is solely based on PAs are available, viz.: (i) pure PAs lure them (Fig. 4A–D; e.g. Pliske et al. 1976; Boppré 1984; Boppré et al. 1984; Boppré and Pitkin 1988; Krasnoff and Dussourd 1989; Dussourd et al. 1989; Häuser and Boppré 1997a, b); (ii) dead or injured PA-insects (Bernays et al. 1977; Boppré 1981; Tea et al. 2021) or pinned individuals in a collecting box (Fig. 4G and H) and faeces of larvae that feed on a PA-plant can attract adult PA-insects; (iii) our unpublished experiments (performed in confinement) showed that PA-plants that do not co-occur with a given insect in the same habitat are attractive, i.e. plants only occurring in Asia or Africa are attractive to insect species that are only found in South America (Fig. 1I) and so on.

Surely, insects visit dry PA-plants solely for PAs; other substances and visual stimuli are not necessary to locate sources of PAs. Strictly speaking, we are not dealing with a relationship between organisms—and certainly not with a typical insect-plant relationship—but rather with an insect-chemical relationship, an association of insects with a certain type of chemicals that is typically provided by plants but which may also be taken up from any other substrate. PA-pharmacophagous insects specifically acquire PAs but are not dependent on a specific source.

### PA-pharmacophagy

Gathering PAs exemplifies the syndrome of “pharmacophagy”: “Insects [animals] are pharmacophagous if they search for certain secondary plant substances [natural products] directly, take them up and utilise them for a specific purpose other than primary metabolism or merely host recognition” (Boppré 1984). While this definition focuses on a common denominator, functional details are manifold and cannot be generalised. A “model PA-insect” does not exist.

PAs are not substances used for primary metabolic processes, and PA-pharmacophagy represents a peculiar behaviour where adult insects gather PAs independently of and in addition to their nutritive requirements—an activity that is quite special. PAs are not necessary for living but potentially promote survival and biological fitness. Actually, PA-pharmacophagous insects do not “feed on” but rather “take up” PAs. Colloquially expressed, adult PA-insects use PA-plants (their secondary/supplementary hostplants) as “pharmacies” for “drug collection”; in this sense, plants that provide nutrients for larval development and flowers or fruit that provide nutritious nectar for adult metabolism (their primary hostplants) represent “groceries”. This distinction is the key to understanding the peculiarity of this unusual type of relationship (see SI 1); in the literature, unfortunately, gathering PAs often is called feeding, thus leading to confusion. Although both nutrients and PAs are ingested via the mouthparts, different behaviours are responsible for locating sources of food and drugs, respectively, and they involve different motivations and adaptations, e.g. as regards sensory receptors.

PA-pharmacophagous insects require both primary and secondary hostplants that are generally taxonomically unrelated and exhibit a dual insect-plant relationship. There are additional implications, including some that had not yet been recognised when the term pharmacophagy was coined.

Insects that pharmacophagously gather PAs are typically neither parasitic nor mutualistic; since the plants used are withered or injured, their fitness is not affected (see SI 1). With PA-pharmacophagy, plants’ defence chemicals are reused/upcycled by insects for their own benefit. This can be viewed as a case of commensalism: for one of the organisms involved, it is beneficial (fitness-boosting), while the other is not affected. Certainly, it is not strictly a case of coevolution as sometimes stated (e.g. Edgar et al. 1974; Edgar 1983, 1984; Livshultz et al. 2018), since PA-pharmacophagy does not involve a reciprocal relationship of species putting selective pressure on each other; rather, the evolution of PAs is driven by guilds of plant parasites, including large herbivores.

The additional behaviour of pharmacophagous insects is ‘expensive’: it can be time-consuming to find a PA-source; it requires energy and exposes the insects to antagonists. The application of fluid to dissolve PAs in dry plant matter (from which a fluid can be re-imbibed only in part) adds to the costs, all of which appear to be worthwhile because the insects become protected against many invertebrate and vertebrate antagonists. This does not exclude that some predators and parasitoids are adapted to consume PA-insects (e.g. Benn et al. 1979; Rossini et al. 2000; Hartmann et al. 2003; Boppré 2008; see also Macel 2011). For the males of several species, PAs represent essential precursors to the production of male pheromones that can increase the chance of mating, which is another benefit of PA-pharmacophagy.

Additional peculiarities of general relevance are associated with PA-pharmacophagy. It is not always a trait of a species but often of one sex only; in many species, only the males are attracted to PAs, while in others, both sexes or only females are (see SI 7). Furthermore, PA-pharmacophagy intrinsically creates individuality among the specimens that form a population: the degree of an individual’s defensive endowment depends on its success in finding a PA-plant and the amount of PA it gathers. Consequently, there is high individual variation in defensive quality within a population, and this is dynamic over time: an adult that has just hatched from a pupa is defenceless, then it gains defensive potency by visiting PA-plants; a male may transfer PAs via its spermatophore to a female and then recharge by gathering more PAs. Females gain protection through this “nuptial gift” from males, part of which they transfer to eggs for their protection (e.g. Dussourd et al. 1989; Brown 1985; Eisner et al. 2000; Trigo 2008). When males synthesise courtship pheromones from PAs gathered by adults, there is individual variation in pheromone amount and consequently in a male’s courtship success, too. The use of PAs for defence and as sexual signals interlaces interspecific with intraspecific chemical communication.

Since PAs are chemicals that are stored, not food that is digested and needs to be regularly supplied, PA-pharmacophagous insects require only a certain amount, i.e. they visit PA-sources only until they are satiated; once they have accumulated sufficient PAs, they stop searching—they do not spend all their lifetime visiting PA-sources. Obviously, individuals decide when to forage for food and when to search for PAs or a mate. How much PAs an individual requires varies between species, considering the great differences in body size of different species. The efficiency of PA-uptake is also variable; it depends on the effort of the insect and the concentration and accessibility of PAs, including the structural characteristics of the plant tissue. A big question is: how does an insect measure the amount of PAs it has acquired—how does it ‘know’ that it has accumulated enough PAs?

In this context, individuality represents a different meaning compared to how it has so far been discussed: the proportion of individuals of a given population that visit a PA-source can vary greatly. Species encountered on PA-baits in small numbers are not necessarily rare, i.e. occurring in low abundance. In some species, the urge to visit sources of PAs may vary between individuals, making PA-pharmacophagy a plastic trait, exhibited individually only under certain conditions (see Lawson et al. 2021).

Table S2 lists the currently known taxa of PA-pharmacophagous insects. Figures 1 and 4 illustrate that PA-pharmacophagy has polyphyletic origin and indicate diversity in size, colours, and lifestyle.

Pharmacophagy is not restricted to PAs; many parallels occur, for example, among insects of different orders that pharmacophagously gather cantharidin, a terpenoid natural product, from certain beetles (Young 1984; Dettner 1997; Hemp and Dettner 2001; Hashimoto and Hayashi 2014). Other cases of pharmacophagy are less complex (see SI 1).

### Insects associated with living PA-plants as food sources

An unknown but definitely large number of insects of various orders are parasites of living PA-plants and use them as primary hostplants—i.e. as food—mostly for larval development (see SI 2). Some of these species sequester PAs and thus are also PA-insects, but of a different kind than pharmacophagous PA-insects. They show relevant parallels with PA-pharmacophagous adults but exhibit more or less high specificity to certain PA-plants and often do not use PAs but other PSMs as stimuli; thus, they remain outside the focus of this paper.

## What is PA-baiting good for?

Knowledge obtained from PA-baiting provides the basis for taxonomic, ecological, evolutionary, chemical, and physiological analyses and puts the peculiarities of PA-insects in general contexts. PA-baiting is needed to obtain comprehensive data and knowledge about the richness of species that are associated with PA-plants as imagines, their biology, and the full range of PA-plants they visit, i.e. the natural sources of PAs for these insects. Even the relationship of one of the most studied and most famous butterfly species, the monarch butterfly (*Danaus plexippus* (Linnaeus, 1758)), with PA-plants remains under-researched (Lawson et al. 2021; Boppré et al. 2022). However, not all questions about PA-pharmacophagy can be answered with PA-baiting alone—experimental and laboratory studies are also required for several aspects (SI 8).

### Taxonomy, species richness, and diversity

PA-baiting is a means to capture insect species that are rarely collectable by other means and find yet undescribed ones. Selected examples demonstrate the potential of PA-baiting: (i) Five of 17 species of *Amerila* (Lepidoptera: Erebidae: Arctiinae) found on PA-baits in Kenya (East Africa) were undescribed (Häuser and Boppré 1997b); *Amerila* are distributed throughout the Old World tropics. (ii) Species of *Digama* and *Aganais* (*Aganais* is now *Asota*; Lepidoptera: Erebidae: Aganainae) were observed on dry PA-plants in SE Asia and PA-baits in Africa (Morrell 1955; Boppré 1999; Sourakov and Emmel 2001); these genera occur widely in the Old World tropics and comprise dozens of species. (iii) During a few visits to two habitats in Kenya, 13 of 18 species of *Gabonia* and *Nzerekorena* (Coleoptera: Chrysomelidae) collected from PA-baits were new to science (Scherer and Boppré 1997); *Gabonia* occur all over Africa with about 100 described species—are all of them associated with PA-plants, and how many new ones may still be found with more PA-baiting? (iv) Recognition of PA-pharmacophagy in African *Zonocerus* grasshoppers has led to the development of a concept to manage their pest populations (Boppré et al. 1984; Fischer and Boppré 1997); are *Z. variegatus* (Linnaeus, 1758) and *Z. elegans* (Thunberg, 1815) worldwide the only orthopterans that exhibit PA-phamacophagy? (v) Numerous Arctiini cannot be collected at artificial light but on PA-plants. Undescribed species, perhaps including further insect orders, may turn up on PA-baits exposed in different habitats during different seasons, particularly when a variety of PA-plants are used as baits (see SI 6, Table S2).

### Habitat evaluation and conservation

Although the number of species known to be PA-pharmacophagous is already large (Table S2), countless habitats have never been investigated, studies are patchy, and the respective assemblages are not fully known.

Considering Arctiini moths, our studies clearly indicate that PA-baiting should not be neglected in the context of inventories, habitat characters, and conservation. For example, out of 216 species of Ctenuchina and Euchromiina that were collected during a long-term study in Panguana (Peru), 49 (23%) were attracted to PA-baits but never by light, 64 (30%) visited light and PAs, and 103 (48%) visited only artificial light; that is, 113 species (52%) could be collected with PA-baiting (Monzón et al. unpubl.). Without giving details, Grados et al. (2015) collected 97 species of Arctiini on *Heliotropium* baits in Cerro Cuchilla (Peru), 48 (50%) of which were not found at light, i.e. 17.2% of the known local Arctiini. Both these cases show that baiting with PAs significantly complements inventories obtainable by collecting with artificial light, but any current numerical evaluation suffers from the heterogeneity of data collection and the lack of good numbers of individuals and must be considered provisional. For inventorying ithomiine butterflies, PA-baiting works well, too (e.g. Masters 1968; Lamas and Pérez 1981; Brown 1985; DeVries 1987; Araújo 2006).

### Evolutionary biology

PA-pharmacophagy definitely has polyphyletic origins but how often did it evolve? Do PA-pharmacophagous taxa share certain ecological or morphological characters? What are their relationship to species whose larvae feed on PA-plants? These and other questions on phylogeny can only be addressed once a more comprehensive list of taxa related to PAs is available. Publications that discuss the evolution of relationships between arctiine moths and PA-plants (e.g. Wink and Nickisch-Rosenegk 1997; Weller et al. 1999; Zaspel et al. 2014) as well as efforts to untangle the phylogeny of Arctiini (Zahiri et al. 2012; Zenker et al. 2017; Dowdy et al. 2020) suffer from incomplete inclusion of PA-pharmacophagous taxa. Early thoughts on the evolution of utilisation of PAs (Edgar et al. 1974; Pliske 1975a; Boppré 1978; Edgar 1982, 1983, 1984) focus on Danainae and included sequestration of cardenolides (based on its understanding at that time, which is now outdated) or do not fully consider the diversity of the syndrome.

Males of many but not all PA-pharmacophagous Lepidoptera possess androconial (scent-producing and scent-disseminating) organs and use PAs as precursors of courtship pheromones, while others use de novo–produced ones; this is another mystery from an evolutionary point of view. More knowledge about PA-insect guilds will facilitate morphological, behavioural, and chemical analyses to elucidate the diversity of PA-derived pheromones in combination with sound evolutionary considerations.

### Mimicry

Although PA-insects can be cryptic in appearance, many exhibit aposematic traits (Figs. 1 and 4), a general indicator of unprofitability to predators (De Solan and Aubier 2019). Many species appear to be involved in Müllerian and/or Batesian mimicry while others are cryptic. PA-pharmacophagy adds new facets to the discussion of mimicry (see SI 1), and PA-baiting can provide relevant information including defence with PAs, diurnal vs nocturnal activity, and aposematic patterns.

## Basics of PA-chemistry and PA-producing plants

### Pyrrolizidine alkaloids

1,2-Dehydropyrrolizidine ester alkaloids (PAs; Fig. 2A–E) represent a major group of PSMs with immense structural diversity (see SI 3). The common denominator of PAs is a heterocyclic necine base (Fig. 2F–I) esterified with one or two necic acids, leading to monoesters, diesters, or cyclic esters (Fig. 2A–E). PAs that are relevant in an entomological context possess retronecine or heliotridine (enantiomers at C7; Fig. 2F and G) as necine bases and occur as free bases (tertiary alkaloids) and as *N*-oxides; both forms (Fig. 2A and B) are interchangeable and can occur together. In living plants, PAs occur mostly as water-soluble *N*-oxides; in seeds, free bases may dominate.

PAs are non-volatile molecules that in living plants are concealed within cell vacuoles. Thus, they cannot be detected from a distance by the olfactory receptors of animals; contact (gustatory) chemoreceptors can, however, taste PAs. Humans regard PAs as bitter. If PAs are not odorous, how can they attract insects from a distance? Current understanding is that drying, like injuring, causes cell damage, and subsequent hydrolysis gives rise to volatile derivatives of the necine base that adapted insects can smell (details in SI 3).

**Fig. 5:**
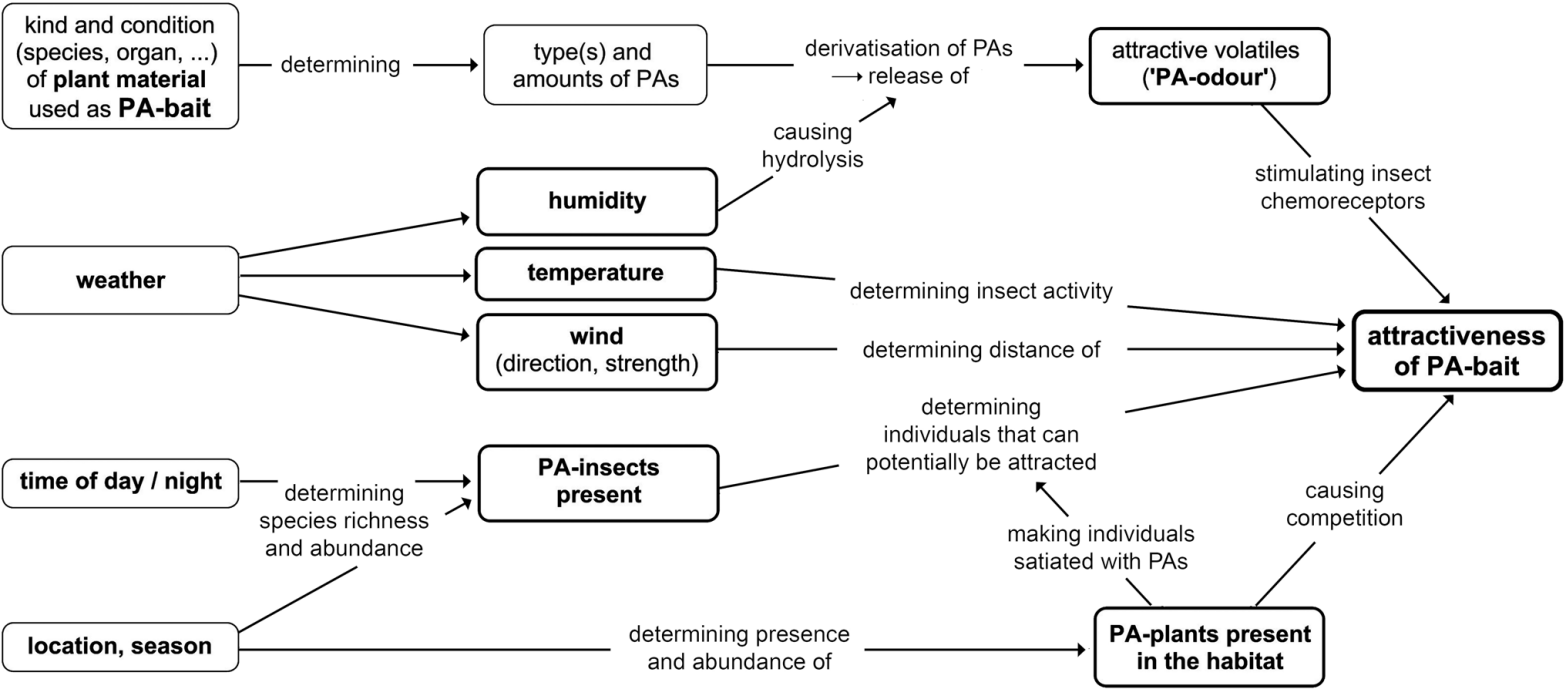
An overview of major factors that generally affect baiting insects with pyrrolizidine alkaloids (PAs). Since, unfortunately, most of these factors vary and cannot be determined or influenced, PA-baiting cannot be fully standardised

Baiting with PAs thus requires humid conditions that cause degradation of PAs and release of a “PA-odour” (Fig. 5), an essential difference from attraction to sex or aggregation pheromones (Plimmer et al. 1982) as well as from the countless plant volatiles that attract insects for egg-laying or gathering nectar (e.g. Metcalf and Kogan 1987) in which derivatisation is not a prerequisite for attractiveness and where systemic volatiles act as indicators for target substrates.

### PA-plants

More than 600 plant species in four families are currently known to produce PAs (Bull et al. 1968; Mattocks 1986; Rizk 1991; Hartmann and Witte 1995; Tamariz et al. 2018; JECFA 2020). Species that are known to cause intoxication of livestock (e.g. *Senecio*, *Crotalaria*), suspect herbal medicines and various food and feed items have been chemically tested for the occurrence of PAs; only relatively few other species have been investigated. Thus, many more PA-plants may still be found, and PA-insects are biodetectors to recognise them (see SI 7).

Taxonomically, PA-plants are found among the Apocynaceae (Echiteae), Asteraceae (“Compositae”; Eupatorieae, Senecioneae), Boraginaceae (Boraginoideae, Heliotropoideae), and Fabaceae (Crotalarieae) (see Table S3). The taxonomic diversity of PA-plants is paralleled by their diverse appearance and lifestyles (Fig. 3): there are tropical and temperate annuals as well as perennials; they grow as shrubs, vines, or even woody trees, and many are ruderals.

Typically, a plant does not contain just a single PA but a bouquet of several structurally different PAs. Bouquets differ most between families and there is a chemotaxonomic pattern (e.g. Hartmann and Witte 1995). It is not specifically known which component(s) plays which role in insect attraction. To date, only relatively few PA-plants have been tested for their attractiveness to insects (see Tables 1, S1, S3, and SI 4).

Within a species of PA-plants, there are genotypic as well as phenotypic differences in the PA content (Hartmann 1996), based on where a plant grows, its developmental stage, and the season; however, not many studies deal with variation. For example, in *Senecio riddelii*, the PA content varies between 0.18 and 17.99% dry weight (Johnson et al. 1985); in *Crotalaria spectabilis*, the concentration of the PA monocrotaline in seeds was found to be 123 times higher than in leaves (Scupinari et al. 2020; see also Johnson and Molyneux 1985; van Dam et al. 1995; Gardner et al. 2006; Eller and Chizzola 2016; Brown and Trigo 1994). In some species, including *Heliotropium*, the whole plant contains PAs; however, within an individual plant, the qualitative and quantitative composition of PAs may vary significantly between organs and over time. Often, PAs are restricted to certain organs such as roots or seeds. In a strict sense, referring to a species as a PA-plant is often not correct because only parts of it may contain PAs; this fact is of great relevance for PA-baiting. Large differences can occur between closely related species (e.g. Williams and Molyneux 1987); in *Crotalaria*, PA-biosynthesis depends on rhizobial bacteria (Irmer et al. 2015). In summary, there is little that can be generalised or predicted as regards structures and quantity of PAs in a given plant, which poses a major issue for PA-baiting.

## The practice of PA-baiting

In principle, baiting with PA-plants is a straightforward and simple exercise. Early reports suggest uprooting a *Heliotropium* plant, hanging it up and collecting the attracted insects for several days (e.g. Hagmann 1938; Moss 1947). Often, within minutes, dozens of insects visit a bait. However, this technique can also be much less successful or even fail.

The many options for variation and, particularly, the limited knowledge on ecologically relevant details in PA-chemistry (composition of bouquets, distribution within tissues, PA-odour, degradation) indicate that only general but not explicit predictions for PA-baiting can be made which leaves some uncertainties. These, however, may not discourage because we have a good idea of the influencing factors. In the following, we provide tips for best practice.

Figure 5 summarises the general factors that determine baiting success and shows that the attractiveness of PA-baits is highly dependent on the context. Note that to set up PA-baits means to put out chemicals that also occur in the environment, i.e. PA-baits potentially compete with naturally present PA-plants. It should also be borne in mind that insects require different amounts of PAs and that individuals become satiated. As such, lured specimens reflect the presence of the species in the area, but species rarely or not found on a bait are not necessarily rare or absent. For conclusions on guilds of PA-insects and their population sizes, long-term PA-baiting is required.

### Plants for PA-baiting

Table S3 provides information on plants that have been found to attract PA-insects or successfully used as baits. Current knowledge suggests that any plant that contains retronecine- or heliotridine-based 1,2-dehydropyrrolizidine ester alkaloids provides good baits when withered or injured. Suggestions regarding plant species to be used as baits have their limitations as there may be differences in the range of insect taxa attracted (see SI 3, SI 6).

Most insect observations were made with species of *Heliotropium* (Tables 1 and S1), and most of the currently known PA-insects have been found on this genus. Pantropical *H. indicum* is one of the most common PA-plants; it occurs seasonally on disturbed soil (wasteland, roadsides, wet fields). Currently, species of *Heliotropium* (Fig. 3A–C and E) appear as a kind of universal joker plant ideal for baiting, although this claim of idealness might be premature; it might be that there are PA-plants with higher attractiveness or with a different range of taxa attracted (see SI 6). Only a few other plants have ever been tested but never extensively.

*Chromolaena odorata* (Fig. 3O) is one of the worst terrestrial invasive plants in the humid tropics and subtropics; it is native to Central America but invasive in Asia and Africa (Zachariades et al. 2009). The highest concentration of PAs occurs in the roots, as is the case in *Emilia* spp. (Fig. 3I–K) and *Erechtites hieraciifolius* (Fig. 3G and H), which also have a widespread occurrence. Roots are typically not accessible to adult insects but can be excellent baits. Species of *Crotalaria* (Fig. 3L–N) are a good option too (SI 7). The genus is common and includes many species (with highly variable PA content), and some are used as crop plants.

A peculiar plant for baiting is *Gymnocoronis spilanthoides* (Fig. 3P and Q; USDA 2017). This aquatic species from South America is sold in many countries (outside the European Union) as an ornamental for aquaria—it is vastly growing and easy to cultivate and propagate (Minolli 2019)—but it is environmentally harmful in foreign habitats. Its roots were accidentally found to be attractive and rich in PAs (Krauska 2009; Boppré and Colegate 2015).

### Durability of the attractiveness of PA-baits

The attractiveness of PA-baits declines over time, with the rate of decline depending on given environmental conditions but also on characteristics of the relevant plant tissue. Typically, a bait should be attractive for at least a week. When its attractiveness fades, shredding or moistening it with an atomiser can reinvigorate the attractiveness, although making a plant properly wet can wash out water-soluble PA *N*-oxides. When baiting in a habitat with low humidity, plant material should also be moistened to make it lure. Instead of spraying water onto a bait, plant material can be placed on top of a wet sponge to boost the humidity in its immediate vicinity.

### Storage of PA-plants while preserving their attractiveness

Since the attractiveness of plants fades under field conditions, newly withered or injured plant material is needed for PA-baiting. However, PA-plants are often unavailable where and when they are wanted. Attractiveness can be preserved for months if plants are stored under dry conditions. Accordingly, plants should be collected whenever the opportunity arises, dried completely (at < 40 °C), and kept in air-tight containers or vacuum-packed in appropriate plastic bags for storage. When plant material preserved in this manner is exposed in the field, ambient humidity typically suffices to make it attractive (i.e. causes PAs to hydrolyse). Collection and storage of PA-plants when they are available allow the exposure of different plant species for comparison. Care must be taken not to leave behind any viable plant materials in a foreign habitat. Note that the attractiveness of freshly collected plants can be prompted by abrading or scratching thick roots or leaves with a knife to damage plant cells.

Pure PAs can also be used as baits (Fig. 4A-C), but only a few PAs are commercially available, and they are expensive (see SI 3).

### Sensitivity to PAs

The maxim “the more, the better” does not apply to PA-baiting. It is not the quantity of plant material but its quality that counts—unfortunately, the quality of PA-plant specimens cannot be fully predicted. Small parts of a dry plant can attract many PA-insects (Fig. 1A–F), even a PA-storing insect pinned in a box (Fig. 4H). The high sensitivity of PA-insects to PA-odour is also obvious when insects visit an empty cardboard box previously used to carry PA-plants or a table on which baits were prepared. Sometimes, even clothes that had been in contact with the plant material can lure PA-insects. Although minuscule amounts of PAs not always cause attraction, it is advisable to avoid contaminations when handling PA-plants.

### Exposure of baits

The most convenient way to conduct PA-baiting is by using a homemade or commercially purchased butterfly trap baited with PA-plant material instead of fruit or fish (Rydon 1964; Platt 1969; Austin and Riley 1995; Hughes et al. 1998; DeVries et al. 2016). However, traps are only efficient for collecting PA-pharmacophagous butterflies (Fig. 4L); other PA-insects, such as moths and beetles, also enter but most or even all will exit the traps eventually. Nevertheless, an initial survey with a butterfly trap can establish whether it is worthwhile to check a bait frequently. A novel bait trap (Fig. 4M and N) with tubes as passages that permit entering but hinder insects from exiting (Monzón, Freitag and Boppré, unpublished) seems promising but requires further testing and optimisation. A properly functional trap will make it possible to compare the ground and the canopy and permit to study several habitats in parallel.

Typically, plant matter should be exposed in gauze bags (Fig. 1G), hung up at eye level in a shaded place where the wind can reach, and protected from rain with a hood (Fig. 4M–O). A gauze bag facilitates the collection of attracted specimens and prevents loss of plant matter. Insect visitors may arrive at any time of the day or night but some species seem to gather PAs at a specific time. Early mornings, late afternoons, and the first 3 h after sunset seem best for baiting. Moderate rainfall does not influence attraction. Insects find odour sources by flying upwind (Cardé 2016); the direction and speed of wind will determine the distribution of volatiles from a PA-source and the distance within which individuals are attracted. A good approach is to hang up several baits along a transect in moderate wind, so that odour plumes can spread over a large area. It is also advisable to place baits at some distance from each other, preferably with plant material of different species, different sources, and different ages, in order to compensate for unseen differences between baits and the dynamics of the plants’ attractiveness. Using sticky material in combination with plant baits is destructive; however, it can be fair for initial testing.

From our experience, baits near forest edges attract more species and specimens than those placed inside dense vegetation; this is likely related to the distribution of odours by the wind. However, species that live only inside dense vegetation need to be baited there. It is not only the quantity but also the variety of captured insects that determine baiting success.

Often, insects land in the vicinity of a bait, then walk and palpate the substrate with their antennae and/or probe it with their mouthparts, where gustatory receptors are located, eventually settling on a ‘good spot’ to apply fluid and extract PAs. On a large bait surface, it can be observed that where a lepidopteran has wetted a spot, others will cluster (Boppré unpubl.; Pliske 1975a; Larsen 1986)—that is, settled butterflies seem to act as visual stimuli sensu “local enhancement” (Otis et al. 2006; Slaa and Hughes 2009). Although visual cues might facilitate finding PA-sources in diurnal species, they are not mandatory.

Several reports state that insects on a bait are kind of doped and can be picked up by hand. This is true only for individuals that are already ‘established’, i.e. those that have deposited solvent. Specimens approaching a PA-source or in search of a spot to apply solvent are typically very sensitive to disturbance and often fly off when a person approximates a bait at a distance of 1–2 m. Insects visiting PA-baits at night, if not yet engaged in extracting PAs, are easily disturbed by torchlight. A red light, which cannot be detected by insects, should be used.

The time insects spend on a bait varies and is unpredictable; some engage in extracting PAs for several hours, while others leave a bait after some minutes. When many insects are on a bait (Fig. 1), they can interfere with each other, causing some to fly away. Regardless, baits should not be left unattended for long—individuals may become satiated with PAs and leave the bait. Checking PA-baits regularly at intervals of approximately 20 mins and collecting specimens with a small transparent container (a specimen tube with a foam stopper) is recommended.

### Desired data records

Records should consider the wider context and not be limited to date, locality, and codes or names for species. The sex of attracted individuals is important for functional interpretations; available data on sex bias is incomplete and, in part, inconsistent (SI 7). Details of used baits (species, organs, condition, source) are necessary. Do visits take place during the day and/or at night? Notes regarding the time may be relevant because some species seem to have typical time windows for exhibiting PA-pharmacophagous behaviour. If PA-baiting is repeatedly poor or very good, not because of the quality of the bait but because of environmental conditions (altitude, season, temperature, region), it is worth recording. In general, documenting the attraction of a certain species or the attractiveness of a given plant/bait is straightforward; however, demonstrating non-attraction or non-attractiveness is challenging. Photographs are always helpful; however, mostly they are insufficient for proper taxonomic identification.

## Incidental observations and follow-up testing

To date, PA-pharmacophagous insects have been observed in the field on a small number of plant species only, and most baiting has been done with plants that were not collected at the baiting site. For example, many species were found on *Heliotropium* baits in forest habitats where *Heliotropium* does not grow. This raises a question of particular relevance: which plants serve as natural PA-sources for PA-pharmacophagous insects?

All the early observations but also several recent papers demonstrate that simply being observant in the field will occasionally result in counterintuitive sightings, such as a butterfly with its proboscis extended on a withering leaf or a wound of a plant. Presumably, many people have made such observations, but not knowing about the context, they were only rarely published (see type I in Tables 1 and S1). However, such incidental observations are important. Ideally, they should be followed up with simple experiments: collecting parts of relevant plants (foliage and roots), drying or injuring and exposing them at different sites during the day and at night, and checking if the original observation is reproducible and whether additional insect species are attracted. In this way, plants can be “biologically identified” as producing PAs and subsequently analysed chemically. This will increase knowledge on plant systematics and toxicology and lead to a better ecological understanding of insect-PA relationships. Several PA-plants have already been discovered with the aid of PA-insects as biodetectors (see SI 7); many more are likely to follow.

Presumably, observations of insects performing PA-pharmacophagy in the field and not on a bait can be made only by chance, not intentionally or by design. To increase the chances of finding natural sources of PAs, researchers should uproot herbal plants, break vines or twigs of bushes and trees, or injure plant tissue (preferably members of families known to contain PAs; Table S3), and then check on successive days and nights for insect visitors.

When visiting remote tropical habitats, making enquiries from the locals (“Have you seen insects on dead plant parts?”) can be rewarding. Numerous PA-plants are traditionally used as herbal medicines, and the local inhabitants may well have noticed insects on them. Information from indigenous peoples can then be followed up in the same way as a researcher’s own incidental observations.

Only a few PA-plants seem to have PAs in their floral nectar (see SI 5). It would be interesting to find flowers that are not attractive to general nectar foragers but specifically to PA-insects; the leaves and roots of such plants should be tested for use as baits. When insects suck and scratch the tissues around open and closed flowers or at buds or withered inflorescences, PA-pharmacophagy is likely.

It is also worth looking out for larvae that feed on living plants of species that are attractive to adults when dry and recording their details. As stated above and in SI 2, many insects use PA-plants for nutritive purposes; they represent significant elements in the ecology of PAs but there is scant knowledge about them. Accordingly, information on larval hostplants of PA-pharmacophagous insects will allow the breeding of selected species and the study of physiological and behavioural aspects.

Assistance in finding PA-plants naturally used by insects can be obtained through internships for biology students, tourist projects at ecolodges, and citizen science projects. Observing insects on PA-baits in the course of edutainment activities can help to foster environmental literacy: people can take photographs of beautiful insects, observe their behaviour, learn that insects have needs besides feeding and mating, and gain awareness of aposematic colouration, chemical communication, and much more; at the same time, they can contribute to scientific research. Many live butterfly exhibits (Boppré and Vane-Wright 2012) house PA-pharmacophagous butterflies (including species of *Danaus*, *Tirumala*, *Euploea*, *Tithorea*, and *Greta*); these locales can also gain from displaying PA-plants.

Laypersons who have been made aware of the topic can contribute by seeking out PA-plants or performing PA-baiting. “Lucky accidents” will increase when more people are involved. Entomological clubs and societies can form groups to study PA-pharmacophagy in their local habitats and researchers can support each other and gather complementary data. When collecting moths with artificial light, PA-baits can be concurrently set up; however, they should be positioned outside the illuminated area because many nocturnal PA-pharmacophagous species shy away from light.

## Perspectives

It is both frustrating and exciting that so little can be generalised about PA-insects, PA-plants, and PA-chemistry, and this will persist because diversity, variation, and complexity are at the centre of the subject. Although a precise manual for PA-baiting cannot be given, the available knowledge is sufficient for employing PA-baiting as a potent means of obtaining valuable data at various levels. PA-pharmacophagy is only one aspect of the complex ecology of pyrrolizidine alkaloids, but it is a significant one that deserves more attention. PA-baiting and scientific natural history studies can address many open questions. Whoever believes that natural history studies including baiting insects are old-fashioned or inappropriate in our digital era should consider, for example, Greene (2005), Berry (2008), Ricklefs (2012), Guidetti et al. (2014), Tewksbury et al. (2014), Ríos-Saldaña et al. (2018), and Cajade and Hernando (2020).

Hopefully, this paper will spread the word on this exciting topic and stimulate entomologists as well as naturalists (due to restrictions of the Nagoya Protocol [CBD 2011] particularly those residing in tropical countries) to make PA-baiting an often-used tool in many habitats and further contribute to the knowledge and understanding of this peculiar type of insect-plant relationship.

### Supplementary Information

Below is the link to the electronic supplementary material.

Supplementary file1 (PDF 2.94 MB)
