# Supplementary material for: Baiting Insects with Pyrrolizidine Alkaloids (PAs): A Fieldwork-Oriented Review and Guide to PA-Pharmacophagy
Source: Neotrop Entomol. 2023 Sep 1;52(5):781–801. doi: 10.1007/s13744-023-01067-9 (PMC10545619; doi:10.1007/s13744-023-01067-9)
Supplement: Supplementary file 1 — Supplementary file1 (PDF 2.94 MB) [file 13744_2023_1067_MOESM1_ESM.pdf]

ad Neotropical Entomology 52 (2023); https://doi.org/10.1007/s13744-023-01067-9

Baiting Insects with Pyrrolizidine Alkaloids (PAs): A Fieldwork-Oriented Review and Guide to PA-Pharmacophagy

Michael Boppré & Julio Monzón

boppre@fzi.uni-freiburg.de

Supplementary Information [1]

The main article characterises PA-pharmacophagy and provides practical tips for PA-baiting according to the maxim "As simple as possible and as complex as necessary", and by referencing the literature for many details. Some generalisations have been made, although the many gaps in knowledge make generalising challenging and, in part, a risky and provisional endeavour. We think a pragmatic approach is justified. The subject is not yet fully understood, but gaps in knowledge add to its fascination as much as the facts do. In the following sections, we discuss some details and additional aspects that would have made the main text too complex but deserve attention.

SI 1: PA-pharmacophagy

The majority of the knowledge on PA-pharmacophagy comes from Lepidoptera. Little information exists about other orders which is clearly due to insufficient investigation (see Tables 1, S1, S2).

In Orthoptera, two species (*Zonocerus elegans, Z. variegatus*) are so far known to be PA-pharmacophagous (Boppré *et al.* 1984). They differ from Lepidoptera in various respects: it is not only the adults but also the nymphs that are attracted to PAs, and grasshoppers possess chewing mouthparts as opposed to proboscides, i.e. they (as well as beetles) are able to gather PAs from living as well as from dead plants and take up not only PAs but also plant tissue containing potential nutrients; thus, a distinction between primary and secondary hostplants is difficult to make. In Lepidoptera that obtain PAs from nectars (see SI 5), it is also difficult to determine if they primarily go for nutrients or PAs.

When Orthoptera and Coleoptera gather PAs from living plants, they act as parasites and can harm a hostplant (see SI 2). Rajesh *et al.* (2012) observed adult *Tirumala limniace* butterflies scratching for PAs with their feet at buds of *Crotalaria retusa* and killing plant individuals in the process; this seems to be an exceptional case and the authors did not provide details.

[1] © 2023 The Authors. This article is distributed under the terms of the Creative Commons Attribution 4.0 International License (http://creativecommons.org/licenses/?lang=en), which permits unrestricted use, distribution, and reproduction in any medium, provided you give appropriate credit to the original authors and the source, provide a link to the Creative Commons license, and indicate if changes were made.

Recently, Tea *et al*. (2021) reported of adult Danaini harassing PA-sequestering caterpillars of *Idea leuconoe* butterflies. It is likely that the caterpillars had been injured and thus became attractive to the butterflies just like plants only become attractive when injured or withered (see main text). Tea *et al*. (2021) have proposed a new term for this relationship: "Since the larvae appear to be unwilling participants in this presumed exchange, we suggest this behavior be termed 'kleptopharmacophagy'." We consider this—as well as their "alternative neologisms kairopharmacophagy (feeding on defensive chemicals from wounded caterpillars detected via "eavesdropping") or necropharmacophagy (feeding on defensive chemicals from dead caterpillars)"—obsolete and confusing, simply because pharmacophagy implies an association of organisms with certain chemicals that are taken from any source. PA-pharmacophagous insects gather PAs not only from plants but also from faeces, dead insects, dishes with crystalline PAs etc (see main text). Many terms that relate to the source but not the target can be coined—but are they any more meaningful?

Despite searching for them intensively, species of *Gabonia* beetles, whose males visit PA-baits in large numbers (Scherer & Boppré 1997; Fig. 4A), were not found in the habitat, except at PA-baits. Their females are unknown; nothing is known about their general behaviour. Males from baits kept in captivity did not survive on living PA-plants, i.e. their primary hostplants seem not to be PA-plants (Boppré unpubl.); they can also be parasitic to PA-plants (see above). The males possess elaborate glands on their antennae, elytra or legs but it is unknown whether they produce PA-derived pheromones or use PAs as nuptial gifts, like many Lepidoptera do.

While, typically, primary and secondary hostplants are taxonomically unrelated, the larvae of *Tithorea* butterflies (Ithomiini) feed on various species of *Prestonia,* which are PA-plants *s.l.* The adults are attracted to various sources of PAs, including the roots of the larval hostplants. Why do the adults go for PAs when their larvae feed on PA-plants? A hypothesis is that certain *Prestonia* possess PAs in the roots only and the larvae cannot take up PAs. Similarly, adults of some *Nyctemera* moths and several *Euploea* butterflies are PA-pharmacophagous as adults although their larvae may feed on PA-plants. Adult behaviour may depend on the plant on which a larva developed. Detailed studies are missing.

Pharmacophagy with chemicals other than PAs

Insect relationships with cantharidin (see main text) is also under-investigated. Research has been conducted sporadically in only a few habitats and for short periods of time, and neither the entire range of canthariphilous species nor all natural sources of cantharidin (particularly in tropical habitats) are known. It is worth noting that cantharidin-seeking insects with chewing or piercing mouthparts might be parasites of cantharidin-producing host species and take up nutrients, too (see above).

Other cases of pharmacophagy are less complex than PA-pharmacophagy and involve single species and/or specific chemicals only (e.g., Nishida & Fukami 1990; Amano *et al.* 1999; Thallamy *et al.* 1999; Nishida *et al.* 2004; Aldrich *et al.* 2016; Singh *et al*. 2022).

PA-Pharmacophagy somehow resembles self-medication (e.g., Abbott 2014), although it is not necessarily related to a disease. In milkweed butterflies, PAs might act as medicine against their protozoan parasite *Ophryocystis elektroscirrha* (Lawson *et al*. 2021; Boppré *et al*. 2022).

Extraction of minerals from mud puddles, dung or carrion (e.g., Downes 1973, Molleman 2010) or drinking of tears from the eyes of vertebrates (e.g., Plotkin & Goddard 2013) is related to food and primary metabolism but shows some parallels to PA-pharmacophagy, too.

Mimicry

Several arctiine moths bear a striking resemblance to wasps (e.g., Moss 1947; Weller *et al*. 2000; Simmons & Weller 2002), including yellow-black bodies morphologically modified to show a petiole-like waist (or colours presenting a pseudo waist) and transparent wings, with the forewings sometimes longitudinally folded at rest. This might not only reflect Batesian/Müllerian mimicry but also masquerade (Boppré *et al*. 2017). Some (but not all) wasp-like moths take up PAs, a detail deserving of further study.

Many more Arctiini moths seem to be involved in Batesian and/or Müllerian mimicry with other moths, butterflies, beetles and other insects (see Simmons 2009), but for most taxa it can only be guessed at who the model and who the mimic or co-mimic is (see also de Jager & Anderson 2019). Faunistic data on insect communities and the biology of the species involved in putative mimicry relationships is largely lacking. Knowledge on PA-pharmacophagy can contribute much to the understanding of chemical defences.

In general, PA-pharmacophagy highlights an issue that to date has been missing in the discussion of mimicry (see Speed *et al.* 2012): biospecies are seen as the entities of models and mimics or co-mimics, but biospecies are defined as a group of individuals that interbreed, not as individuals that share the same characters. The individuality of PA-pharmacophagous insects due to their defensive endowment with PAs (see main text) implies that they comprise ecological entities with individual and even temporally dynamic defensive traits; this goes beyond the issue of automimicry (e.g., Turner 1984; Guilford 1994; Svennungsen & Holen 2007) and requires attention.

SI 2: Parasites of PA-plants

Many insects of various orders use a wide range of PA-plants as primary hostplants, but this aspect of the ecology of PAs has not been studied extensively except for several species that were investigated in some detail (e.g., Rothschild *et al*. 1979; Dobler *et al*. 2000; Pasteels *et al*. 2003; see also Witte *et al*. 1990; Klitzke & Trigo 2000; Loaiza *et al.* 2007; Wei *et al*. 2015).

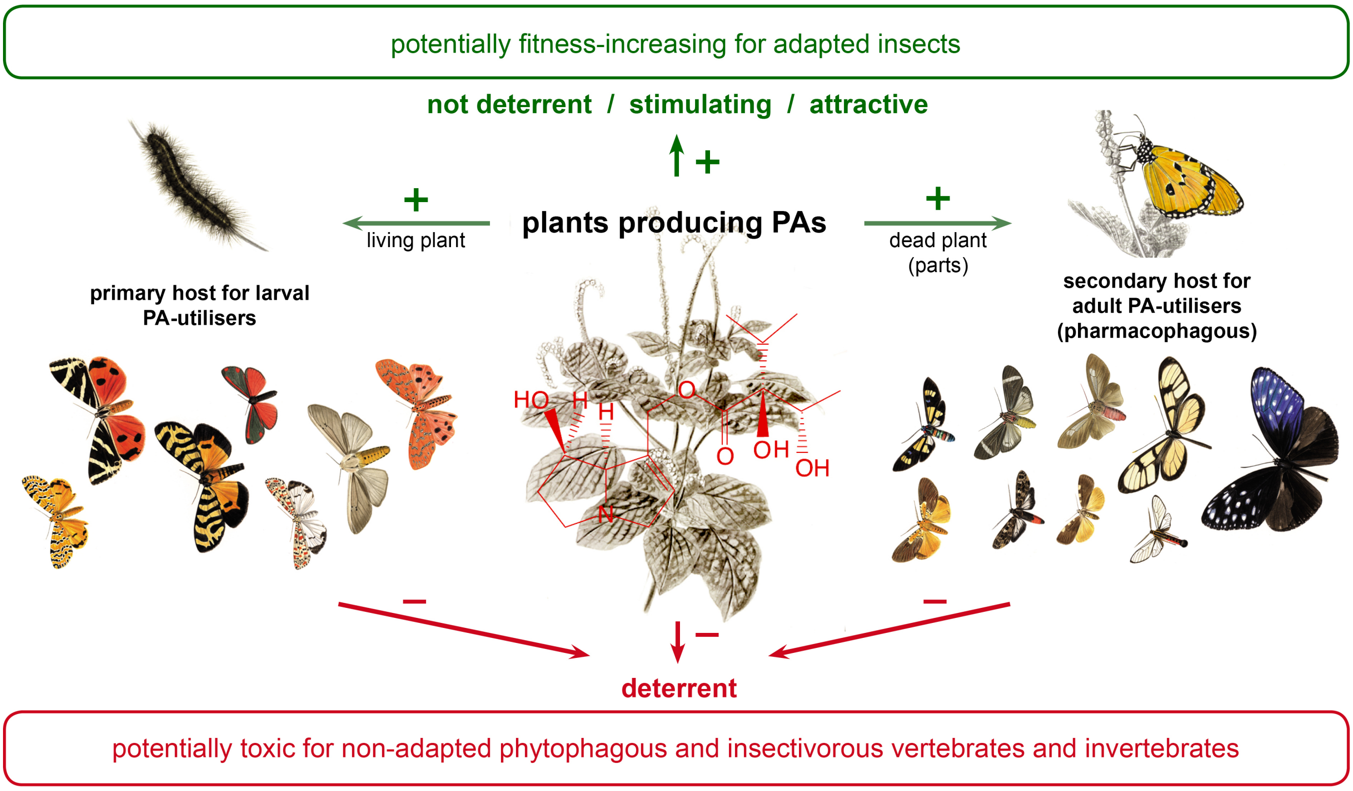

Figure SI 2. PA-insects sequester 1,2-dehydropyrrolizidine ester alkaloids. Some obtain them from living larval (primary) hostplants, while others are PA-pharmacophagous and gather them from secondary hosts as adults. (After Boppré 1995: fig. 15.)

Studies on Lepidoptera show that some degrade or excrete PAs, while others are not only adapted to ingesting PAs without being harmed but can even sequester them for their own defence (e.g., Trigo *et al*. 1993; Boppré 2011: fig. 2; Martins & Trigo 2016). Yet others—including the well-studied *Utetheisa* moths (Eisner 2005; Conner 2009)—in addition use PAs to synthesise male courtship pheromones (Edgar 1972; Schneider *et al*. 1982; Krasnoff & Roelofs 1989; Davidson *et al*. 1997; see Schulz 2009); as in PA-pharmacophagy in such species, PAs interlace intra- with interspecific chemical communication.

Strikingly, many Arctiini seem to produce hydroxydanaidal (Fig. 2K) only. In *Creatonotos* and *Estigmene* (and other species?), PAs also serve as morphogens that trigger the growth of the coremata (male scent organs) (Schneider *et al*. 1982; Boppré & Schneider 1985; Davenport & Conner 2003; Jordan & Conner 2007; Conner & Jordan 2009), which is yet another effect PAs can have for insects although it cannot be generalised.

Many insects that use PA-plants as primary hosts relate to PAs 'unconsciously', i.e. their choice of a hostplant is not mediated by PAs or derivatives thereof but by other PSMs. Their relationship with plants can be more or less specific, i.e. a given species of PA-insects uses one or several specific PA-containing hostplants but not just any PA-plant, or the range of hostplants may also include non-PA-plants.

This is in accord with the majority of insect-plant relationships where insects use and benefit from PSMs (including cardenolides, cyanogenic glycosides, terpenes, and phenolic compounds; e.g., Opitz & Müller 2009).

Again, the details are complex. For instance, the larvae of *Creatonotos* consume glass-fibre filter discs when they have been impregnated with PAs (Boppré 1995); this clearly meets the definition of PA-pharmacophagy, but larvae naturally never have the opportunity to ingest PAs except in combination with food. The cannibalism that Bogner & Eisner (1991, 1992) reported for eggs and pupae of *Utetheisa ornatrix* is also in the context of obtaining PAs.

All species that either feed on PA-plants as immatures and utilise ingested PAs or gather PAs as adults in a pharmacophagous way are PA-insects. Although at first glance no large differences may be observed concerning the functional roles of PAs for the two kinds of PA-insects (see Fig. SI 2), from an evolutionary perspective it is fundamental to make a distinction. It implies various consequences including different sensory and metabolic adaptations, specificity, processing of the chemicals, motivation, etc.

The examples above (even more examples abound, and many more will likely be discovered) demonstrate that studying the relationships between insects and PAs in the wider context of the ecology of PAs is a rewarding field of research, and there is need to further investigate not 'only' PA-pharmacophagy but insect-PA relationships in general.

SI 3: Chemistry of PAs

Currently, more than 600 structures of 1,2-dehydropyrrolizidine ester alkaloids have been identified (e.g., Bull *et al.* 1968; Mattocks 1986; Rizk 1991; Hartmann & Witte 1995; Moreira *et al.* 2018; Tamariz *et al.* 2018; Schramm *et al.* 2019; JECFA 2020), and many more may still be found. Small modifications in PA-molecules such as isomers or enantiomers, although technically discriminable, might but do not necessarily need to be biologically relevant for sensory systems and/or for metabolic processing, i.e. insects might treat certain structurally different PAs as equally (see, e.g., Macel *et al*. 2002). Nevertheless, structurally similar PAs can differ in their effects (e.g., Silva & Trigo 2002). Attractiveness does not reflect an intrinsic character of a chemical but an effect on a receiving organism.

In chemical nomenclature, several PA-like molecules found in orchids or grasses are also called PAs but they have no relevance for insects. PA-pharmacophagy appears to be restricted to 1,2-dehydro-pyrrolizidine ester alkaloids.

Toxicity of PAs

Retronecine- and heliotridine-based tertiary PAs are pro-toxic. Their bioactivation by vertebrate metabolism forms pyrroles that cause (dose-dependently) incurable and often lethal hepatotoxic, mutagenic or carcinogenic effects (e.g., JECFA 2020). PAs provide chemical defence for PA-producing plants since they act as deterrents in a dose-dependent manner and render PA-plants unpalatable for unadapted animals; however, livestock may consume PA-plants when alternative forage is unavailable. Because unadapted insects do not voluntarily ingest PAs, it is difficult to establish whether they would suffer toxic or even lethal effects. Insects cannot suffer from hepatotoxicity but may experience genotoxicity (see Frei *et al.* 1992) or other harm (e.g., Narberhaus *et al.* 2005). Are assays that involve injection of PAs into insect bodies (e.g., Nuringtyas *et al*. 2014) biologically meaningful? Note that adapted insects can enzymatically convert tertiary PAs into metabolically safe *N*-oxides (Lindigkeit *et al*. 1997; Sehlmeyer *et al.* 2010). In any case, PAs provide protection for plants and insects against many vertebrate and invertebrate antagonists although it is not absolute and consumers adapted to PAs do exist.

Many plants that produce PAs are used as traditional medicines (e.g., Roeder 1995, 2000; Roeder & Wiedenfeld 2011, 2013; Dash & Abdullah 2013; Neumann *et al.* 2015; Fu *et al.* 2022). This may not distract from the fact that the pro-toxicity of PAs has detrimental effects and poses serious health concerns. The positive medicinal effects ascribed to PA-plants, in cases in which they have been proven, are due to PSMs other than PAs.

Sensing PAs

PA-molecules are not volatile, but their ester bond(s) are susceptible to hydrolysis (Fig. 2A) which yields the necine base plus necic acid(s) that are both volatile. When plant parts wilt or become injured, cells break open and PAs are exposed to ambient conditions under which they hydrolyse more or less easily, depending on the strength of the ester bond(s) which relates to their overall molecular architecture.

The derivative(s) that make up the odour of PAs ('PA-odour') for adapted insects have, unfortunately, despite many efforts, not yet been identified. Apparently, PA-odour is sensed by specialised antennal chemoreceptors in trace amounts (similar to female sex-attractants) and is likely short-lived because of its vulnerability to oxidation. As the diversity of PA-molecules involves a few necine bases only but hundreds of esterifying necic acids, many of which show only little structural differences, evidence suggests that PA-odour probably represents one or several necine base derivatives, i.e. dihydropyrrolizines, which are similar to pheromones made from retronecine- and heliotridine-based PAs (Fig. 2K–M). Hydroxydanaidal (Fig. 2K)—or a derivative thereof—is a candidate molecule: it attracts insects searching for PAs (Krasnoff & Dussourd 1989; Fig. 5F); there is a dedicated receptor for it (Bogner & Boppré 1989); Edgar *et al.* (1973) could wash hydroxydanaidal from *Heliotropium amplexicaule* after moistening it.

Although it is difficult to imagine a receptor that can detect the diverse acids, receptors for certain necic acids are not ruled out, and some PA-insects may sense them: for instance, ithomiine butterflies are not attracted to some PA-containing *Eupatorium* species (Trigo *et al.* 1996), to *H. angiospermum* and *Tournefortia volubilis* Pliske (1975a), nor to the roots of *Emilia* or *Erechtites* (Boppré unpubl.) that are highly attractive for many other PA-insects. For Ithomiini plants containing monoesters are most attractive (Trigo *et al.* 1996). In contrast to Danaini and Arctiini, Ithomiini produce male pheromone components not only from necine bases but also from certain necic acids (Fig. 2K,N,O; Edgar *et al.* 1976; Schulz *et al.* 2004). It is unknown whether ithomiines generally respond to different volatiles compared to other PA-insects. The findings of Pliske *et al.* (1976) in this regard are doubtful due to the decomposition of their test compounds and require verification. Regardless, hydrolysis appears to be essential in rendering a PA olfactorically detectable but the matter is still open as long knowledge about the reactions of both dehydropyrrolizidines and dihydropyrrolizines under ambient conditions is insufficient.

Techniques for analysing PAs in plant matter are steadily optimised to detect medicinally relevant compounds (e.g., Kopp *et al*. 2020) but the elucidation of full bouquets, including minor compounds and/or derivatives, is not the focus. It may well be that volatile derivatives of attractive PAs are not solely produced *via* hydrolysis but rather are present *per se*: a few studies did find non-dehydro PAs as minor components (boveinine [Reina *et al*. 1998]; loroquine [Borges del Castillo *et al.* 1970]; parsonsine [Abe & Yamauchi 1987], Fig. 2P) that share the characteristics of dihydropyrrolizines (Fig. 2K–M). Are such molecules common in PA-plants but escape detection when extraction techniques tailored to the detection of toxic PAs are applied?

It is not only extraction but also analytical methods that cause a preselection of analysable chemicals; therefore, adequate means need to be employed to find volatile derivates that adapted insects consider attractive. Analysis of head-space air appears to be the method of choice; however, it reveals an overwhelming number of peaks and there are difficulties associated with the selectivity of adsorbents and problems with the desorbing and detection procedures (Boppré *et al*. unpubl.). Another issue involves the quick oxidation of many dihydropyrrolizines (see Culvenor *et al*. 1969, 1970; Edgar *et al*. 1973) which makes chemical characterisation (see Stamm *et al*. 2019) as well as the conduction of bioassays demanding.

Unfortunately, characterisation of the PA-odour, is highly challenging. Bioassays performed in captivity with a 'cooperative' species of PA-insects and a wide range of PA-plants might contribute to finding the best kind of plant material for chemical analyses but even this is difficult to conduct accurately.

Stability of PAs

Much is known about the 'behaviour' of PAs in plant and animal metabolism but almost nothing about their reactivity in ambient conditions. Generally, chemists keep natural products in a fridge and avoid exposing them to conditions that may cause their degradation by oxidation or UV light and temperature. When conducting baiting, conditions are always unique, all the PAs which the bait contains may not be known, and the behaviour of the PAs may not be fully understood. Moreover, the attractiveness of dried PA-plants declines over time (see main text). Then, PAs are still present within the tissue and can be extracted with solvent although they escape hydrolysis and for insects are thus undetectable. For studies on the roles of PAs in ecology, data on reactions of PAs under ambient conditions is needed.

SI 4: PA-plants

There is quite a large number of publications on the chemistry of PA-plants. Understandably, analytical chemists focus on the identification of new structures in natural products, particularly those with medicinal and/or veterinary relevance. Thus, from an ecological perspective, the literature on PAs suffers from significant limitations: i) a general screening is missing; ii) mostly entire plants were extracted, although it is known that PAs are not always distributed equally in a given plant individuum; iii) often only major compounds are structurally elucidated, i.e. we have insufficient knowledge of full bouquets; iv) often PAs are not quantified in detail; v) use of a huge array of extraction and analytical methods provide incomparable results; vi) little information exists on intraspecific / seasonal variation; vii) ongoing taxonomic changes in plant names and viii) inadequate determination as well as ix) inconsistent use of chemical terminology cause confusion.

Many more PA-plants will be discovered, and some may be more attractive than the currently known ones. The majority of analysed PA-plants are from ruderal habitats. Knowledge of PA-plants from tropical forests that are particularly relevant to insect-PA relationships is scant. PA-insects can be employed as biodetectors (see SI 7).

Concerning the systematic distribution of PAs in the plant kingdom, knowledge of the variation and diversity of PAs in plant taxa is complex and incomplete (see SI 3). Simple generalisations are impossible to make at present.

PA-plants each have qualitatively and quantitatively different bouquets of PAs. This makes it impossible to find out exactly which PAs are relevant to the attractiveness of the given plant material to PA-pharmacophagous insects. Only from finding that the insects are attracted to pure PAs do we know that the attractiveness is directly related to PAs and not to any other plant chemicals. Not all PAs can be tested in their pure form; many do not crystallise and, in particular, the uncertainties with respect to their degradation under ambient conditions persist.

SI 5: PAs in floral nectar

A peculiar issue concerns flowers. The nectar of most PA-plants apparently does not contain PAs, which deter insects that are not adapted to them (Masters 1991). However, it appears that there are at least some PA-plants that have PAs in their nectar and, in this way, recruit a specific guild of pollinators:

In Argentina, Jörgensen (1913) wondered at masses of Arctiini (as Syntomidae) visiting flowers of *Senecio brasiliensis* but also injured green parts and wilted stems; this plant was later found to produce PAs (Hirschmann *et al.* 1987; Klitzke & Trigo 2000; Sandini *et al.* 2013). Flowers of some *Eupatorium* spp. also contain PAs in their nectar and seem to be exclusively visited and pollinated by PA-insects (e.g., *E. xestolepis*, Pliske 1975b). Brown (1987) found that Ithomiini gather PAs mainly from flowers; he assumed that PAs are synthesised in the roots and transferred to the inflorescences. Unfortunately, the roots have never been bioassayed or chemically evaluated. In Africa, the flowers of *Gynura scandens* were found to be exclusively visited by *Gabonia* beetles and Danaini, the latter exhibited the same sex bias as at PA-baits (Boppré 1990). In Argentina, Malcolm & Slager (2015) found "thousands" of *Danaus erippus* nectaring at flowers of *Chromolaena arnottiana* (as *E. arnottianum*), which contain PAs. Flowers of *Gymnocoronis* might also contain PAs in their nectar.

PA-insects have often been found on flowers of *Ageratum conyzoides* (Fig. 3F, Tables 1,S1), one of the most common pantropical ruderal weeds that contain PAs (Wiedenfeld & Röder 1991; Bosi *et al.* 2013; Almeida & Ravindran (1988) doubted that *Ageratum* contains PAs but they used inappropriate analytical methods). Close observation shows that PA-insects often do not take up nectar but rather extract PAs from tiny dry parts of an inflorescence. Even if PAs are chemically detected in extracts of inflorescences (Brown 1984, 1987; Trigo *et al.* 1996), this is not conclusive proof that PAs are constituents of the nectar.

Detailed studies are needed to elucidate the question of the presence of PAs in nectar. When flowers are suspected to contain PAs in their nectar, tests with withered flowers as well as other tissues are strictly necessary for verification. Examining the guild of flower visitors may also help; PA-pharmaophagous moths should be included in any such study. Note that, in general, it is difficult to experimentally collect nectar from small flowers without wounding the nearby tissue. Allowing PA-insects to consume floral nectar of inflorescences devoid of wounds and dry parts and analysing the insects for PAs seems like a simple test, but it requires a culture of appropriate insects and plants as well as chemical analyses.

A particularly interesting case involves flowers of *Epidendrum paniculatum, E. anceps* and *E. densiflorum* orchids which appear to be pollinated exclusively by PA-insects although they produce neither nectar nor PAs and thus do not provide a reward; rather, these flowers appear to deceit PA-insects with *de novo* synthesised PA-odour or a volatile that is mimicking PA-odour and which the insects' chemoreceptors cannot distinguish (Wagner 1973; Adams & Goss 1975; Goss & Adams 1976; DeVries & Stiles 1990; Pansarin 2003; Silveira *et al.* 2023; Boppré manuscript). Unfortunately, the observations have not been followed up in a systematic way. Silveira *et al.* (2023) did not study flower visitors at night; our

considerations on volatiles rendering PAs olfactorically detectable for adapted insects (SI 3) put their hypothesis "... the flowers of *E. densiflorum* (and related species) mimic the fragrances of plants that are the actual alkaloid sources for these Lepidoptera" into context. The role of PA-insects in pollination of *Epidendrum* species is another subject that requires more attention. In order to identify the PA-odour, perhaps, the composition of head-space air of fresh *Epidendrum* flowers contains less chemicals than head-space air of decaying PA-plants and is therefore less difficult to analyse.

SI 6: PA-baiting

Specificity of attraction

Most observations on PA-pharmacophagy were made using species of *Heliotropium,* which obviously are prime plants for PA-baiting. Indeed, PA-baiting mostly focusses on *Heliotropium*, although the context with PAs often is not appreciated and other sources of PAs remain unconsidered. Definitely, *Heliotropium* is a genus that guarantees baiting success; however, it is all but justified to assume it attracts the (yet unknown) full range of PA-insects.

Baiting with different plants in parallel whenever and wherever possible is urgently needed on a large scale to learn about the specificity of attraction. That Ithomiini are attracted only to certain PA-plants (see the subsection on sensing in SI 3) might also be true for certain Arctiini and other taxa.

When insects are given a choice between several baits made from the same plant species they may prefer one over the other, but we cannot know whether their choice is due to individual PAs or the bouquet of PAs or the type or amount of volatiles, and it may change over time. When two or more different plants are placed next to each other as baits, the same uncertainties apply, and a quantitative evaluation of such simple choice tests appears impossible. However, choice tests are very informative when different insect species are attracted in meaningful numbers. In general, documenting the attraction of a certain species or the attractiveness of a given plant/bait is straightforward, while demonstrating non-attraction or non-attractiveness is challenging. Information on specificity is highly demanded and might set a milestone for understanding PA-pharmacophagy and its evolution. It makes much sense to continue to use *Heliotropium* as baits in many habitats and in different seasons, but PA-baiting should not be restricted to this plant but be complemented with others.

Commercially available PAs

A few milligrams of pure PAs offered in dishes do attract PA-insects (Fig. 4A–D); however, according to our experience, plant material is usually more attractive than pure chemicals or extracts, although this cannot be fully explained yet. Also, pure crystals can quickly be consumed by insects, and they are expensive. For example, 250 mg of heliotrine from Latoxan currently is EUR 400 (Latoxan 2023), while 10 mg from SIGMA-ALDRICH is EUR 402 (SIGMA-ALDRICH 2023); 20 mg of monocrotaline from Extrasynthese is EUR 112, 10 mg of Senecionine is EUR 186 (Extrasynthese 2023), while 25 mg from Selleck is USD 127 (Selleck 2023). The companies recommend to store PAs at +4 to -20° C!

Habitats for PA-baiting

It is assumed that PA-insects will be found in all kinds of (sub-)tropical habitats. However, habitats likely differ with respect to presence of PA-plants and PA-insects; a qualified statement can only be made when more habitats have been investigated.

See also SI 7 "Conditions for PA-baiting".

SI 7: Comments on selected publications that require verification

Diversity of insects found at PA-baits

Occasionally, a specimen of an unexpected taxon (e.g. pyralid or geometrid moths, cockroaches and other insects) is found on a bait. These insects may visit baits not because of PAs but for other ingredients or contaminants such as salty sweat from handling. Only when a taxon not yet known to exhibit PA-pharmacophagy is found repeatedly and in good numbers on PA-baits is there an indication of a new valid record of a PA-insect.

In this vein, Hagmann (1938) stated: "unpleasing is the fact that fedegoso [*Heliotropium*] attracts mosquitoes (Culicidae) that mercilessly pursue the collector. During the day, the fedegoso lick is frequented by many wasps that gnaw it on the lance, on the leaves and on the panicle and it is mainly in the wounds that form there that the butterflies take advantage of it to suck the fedegoso's sap." Similarly, Jörgensen (1913) found stingless bees (*Trigona amalthea*) and wasps (spp. of *Polistes* and *Polibia* [sic, recte *Polybia*]) biting holes in *Senecio brasilienis* plants and drinking the exuding sap. Large numbers of *Trigona recursa* were observed consuming fruits of *Crotalaria micans* (Santos *et al.* 2013). Moss (1947) found, most remarkably, attraction of drying *Heliotropium* also "for wasps of many kinds, for a few beetles, for grasshoppers, for bugs, mosquitoes and flies of all sorts." Pliske (1975a) collected Orthoptera (Tettigoniidae, Gryllidae), Dictyoptera (Blattellidae, Blattidae), and Coleoptera (Cerambycidae) on PA-baits in Venezuela.

Unfortunately, all these papers do not provide enough details, most do not even give the names of the observed species. Thus, whether the diverse insects reported on baits (or some of them) are PA-pharmacophagous or rather use the plants for food (see SI 2) needs to be verified. None of the mentioned taxa have been found by us in Kenya, Costa Rica or Peru, but it may well be that in certain habitats there are specific wasps and insects of other orders that gather PAs in a pharmacophagous way. Indeed, many new records are quite conceivable and even expected with additional PA-baiting, and further studies in many different regions will enrich our currently incomplete knowledge and perhaps make PA-pharmacophagy an even more significant subject than it is currently is.

Pliske (1975a) also collected Diptera (Chloropidae) on PA-baits. Chloropidae definitely go for PAs because they were also found to be attracted *en masse* to dishes containing pure PAs in Africa (Boppré & Pitkin 1988), Costa Rica and Peru (Boppré & Monzón, unpublished; Fig. 4K); they preferably visit just before dawn and appear a good indicator of the quality of a bait. However, an idea on the role(s) PAs play for them is missing; their taxonomy is challenging.

Incidental observations and follow-up testing

Among the relatively many incidental observations of insects on withering PA-plants several are most

impressive by reporting hundreds of individuals (see Tables 1,S1). Unfortunately, most reports deal only with acute situations, with no follow-up observations (e.g., observations in the following days and nights or testing of plant organs separately and in different habitats) and subsequent chemical analyses. Thus, more in-depth studies need to be conducted on the numerous plants which were found to be attractive when injured or withering.

For example, many field observations were made with *Heliotropium foertherianum* (Tables 1,S1) in the daytime. Apparently, nobody has ever checked this plant for insects at night or in distant habitats. It would be rewarding to take samples (seeds, twigs, bark, roots) as baits to other habitats and check them during the day and at night. *Heliotropium foertherianum* is one of the few PA-plants with a tree habit; thus, it is available all year and widely distributed on the tropical shores of the Pacific and the Indian Ocean. (Chemical analyses [Edgar 1982; Ogihara *et al*. 1997] are insufficient; a modern re-investigation is wanted that considers the full bouquet of PAs and differentiates between organs.)

About 20 publications reported Danaini visiting buds, pods and (rarely) leaves of *Crotalaria* species (Tables 1,S1), mostly in high numbers, however, all observations were made during the day; the attractive plants were never checked at night, and baiting tests (also involving roots) have not been conducted yet. It should be a general rule that plants that were incidentally found to be attractive are kept under observation for some days and also tested in other habitats during the day and at night (as Hagmann [1938] and Zerny [1931] did with great success but with *Heliotropium* only). PA-containing exudates seem unique for *Crotalaria* but this character requires more detailed studies.

Incidental observations of Lepidoptera with extended proboscis on withering or damaged plant parts will lead to greater knowledge on the richness of PA-plant species. So far, PA-pharmacophagous insects have served as bioindicators for finding PAs in some plants (*Echites panduratus*, *Parsonsia* spp., *Gynura scandens, Prestonia amabilis, Chromolaena odorata, C. arnottiana, Gymnocoronis spilanthoides, Alafia* cf. *caudata, Amphineurion marginatum* – see Table S3); several attractive taxa (including *Neomirandea* sp., *Senecio burkartii*, *Adelocaryum coelestinum*) require confirmation of the presence of PAs by chemical analyses.

In general, it would be very helpful if entomologists who engage in PA-baiting reported, in addition to their target species, on other insects they find on baits and on the abiotic context.

Conditions for PA-baiting

Hagmann (1938) states that "absolutely negative [for baiting success] are the moonlit nights"; this contradicts all our experience—we rather enjoy baiting when the moon is shining because our general orientation is facilitated. Several other of the early publications also make statements which we cannot confirm.

Pliske's (1975a) paper, published at a time when the topic of PA-pharmacophagy was still in its infancy, is a pioneering and seminal work. It not only compares several PA-plants but also habitats and

reports and discusses many basic details (including sex bias, attractive cues and functional aspects). Pliske collected 257 species of Lepidoptera and some other taxa but of many only small numbers; considering that he used different plants in habitats with different faunas without stating the baiting durations, a comparative and detailed evaluation seems impossible. However, his records remain highly valuable but some of his conclusions require modification in the light of advanced knowledge.

Ramos *et al.* (2020) concluded from their experiments that "*Danaus* butterflies of the Americas do not perform leaf-scratching", implying that they do not sequester PAs (see also Ramos *et al.* 2019). Their approach needs to be questioned because, unfortunately, they wrongly assumed that "butterfly species and not plant condition serve as the explanatory variables" for attraction to fresh compared to withered (*Crotalaria*) plant material and the "underlying mechanisms that mediate this interaction [PA-pharmacophagy] have not been explored". They also were not aware of and did not consider many other principal aspects related with PA-pharmacophagy and necessarily came up with doubtful results.

Ancajima & Neyra-Hidalgo (2021) used *Heliotropium* baits at 21 spots in Peru, which were checked frequently for three months. They collected 39 specimens of 16 species of Arctiini—a very poor gain for a big effort. Probably, they were not aware of the variables that need to be considered when performing PA-baiting; likely their baits were not in good condition and/or their traps did not retain all specimens attracted. Unfortunately, not enough details are provided in the study's Material and Methods section.

Sex ratio and physiological state of specimens attracted

Some reports (e.g., Pliske 1975a) suggest that PA-insects can be categorised on the basis of which sex is attracted by PA-plants. For a few species it is clear that attraction is sex-biased, however, on most species data are missing and on others too few specimens were checked. Extensive data is much wanted on as many species as possible—it will be important for drawing functional conclusions.

The study of Goss (1979) is the only one which analysed the age (based on fat body) and mating status (based on presence of spermatophore(s)) of specimens attracted to *Eupatorium capillifolium* baits in Florida, USA. Unfortunately, his data is too little to allow for making up categories, however, such physiological data, in combination with data on sex ratio permit more extensive interpretation and should be collected on a larger scales.

SI 8: Epilogue

Although the first observation of PA-pharmacophagy dates back more than a century, we are only at the beginning to understand the syndrome with all its facets. For several decades, counterintuitive sightings of flocks of butterflies sucking at dry plant matter were puzzling, and the occasional taking advantage of the attractiveness of PA-baits for collecting certain arctiine moths was a means that had no explanation. Note that the early authors reported butterflies at PA-plants long before PAs were initially characterised by natural product chemists (see Bull *et al*. 1968).

In recent decades the issue has become a complex subject with many intertwined aspects which made it a topic of Integrative Biology. The interest in PAs as a health risk for humans and livestock resulted in many studies on PA-chemistry and presence of PAs in plants from which entomologists could benefit a lot. The increasing knowledge over time requires 'correction' of some statements made in the literature.

We have tried to draw attention to the relevance of PA-pharmacophagy and untangle and explain the complex context by reviewing and structuring background facts in order to constructively demonstrate the avenue to go.

Much of the information is directed towards a holistic understanding of PA-pharmacophagy. We inform colleagues interested in insect-plant associations, chemical ecology, and ecology, biology, and evolution in general as well as students of scientific natural history about a research topic with great research potential in various aspects. For authors who published observations of insects at PA-plants but obviously being not aware of the context, we provide a framework to accomodate their findings.

For colleagues with a strict focus on taxonomy and faunistic aspects, many details might be irrelevant, however, they can extract the practical tips given. Long before the luring capacity of PA-plants was understood, collectors employed PA-baiting with success, and until today, PA-baiting can be successfull without considering all the context which is now available. However, we think it is helpful to have in mind some principal knowledge on PA-chemistry, PA-plants and PA-insects; if collectors are aware of the factors that influence baiting success and, for example, use plant material of optimal condition and a wider range of plant species for baiting, they will obtain more insect species and specimens—at the same time they can also contribute to general aspects if they publish details of they work.

In particular, the understanding of PA-pharmacophagy suffers greatly from rarity and patchyness of records and that mostly a few specimens only were recorded. The many reports of incidental observations, often made at a single site, on a single day and of a few specimens do not provide robust data although they do stimulate follow-up studies. We plea for PA-baiting at many more sites, using many more plant species as baits, and conducted for longer periods of time to obtain more specimens of species that occur in low abundance as typical for tropical ecosystems.

Individual studies can contribute much, however, since we are dealing with biodiversity *s. str.* what is really required is that circumtropically in as many habitats and at as many times as possible PA-baiting

is conducted to contribute to increase knowledge on the diversity of PA-plants and PA-insects and bring the subject to a higher state of knowledge.

With PA-baiting alone, knowledge on attractive plant species and attracted insect taxa but also on specificity, sex-ratio, mating status etc can be gained. This is a lot but not all. Eventually, contributions by scientists from various disciplines, natural product chemistry in particular, are needed, too. Both fieldwork and laboratory-based studies should be conducted. A comprehensive study of the topic is like a challenging jigsaw puzzle that requires many different players who complement each other.

Several aspects seem easy to investigate, but his is easier said than done. For example, it would be most interesting to investigate the attractivity of the Old World *H. foertherianum* (see above) in Latin America where the diversity of PA-pharmacophagous Arctiini is highest. This is not possible due to meaningful restrictions on the import of plant material. Also, comparison of the use of different pure PAs as baits is supposed to be easy, but pure PAs are mostly unavailable, expensive, and degrade easily.

Many questions could be answered if breeding cultures of several PA-pharmacophagous species in confinement were available; however, we hardly know a valid name for many of these species and only know the larval hostplants for a few. One not only needs appropriate conditions to breed a given insect species but also to cultivate its hostplant, which is an obvious challenge. To have different plants available for baiting at the same time is easy to plan but ensuring the right condition for the plants at a place where many insects are abundant and where there is little competition with natural PA-sources is difficult; one needs to be lucky to be able to perform quite simple tests. The many gaps in knowledge on basic natural history of insects such as hostplant relationships—another field which requires many studies.

Let us end by saying: *You grow with the challenge*. Despite the many uncertainties and limitations discussed, we hope to have motivated many colleagues, particularly those who live in the tropics, to contribute to PA-pharmacophagy.

Table S1

Publications complementing Table 1. Chronology of original reports on incidental observations (column type: I) and experimental bating (column type: E) on insects attracted to sources of pyrrolizidine alkaloids (PAs) that do not show pincipal news and, therefore, are not mentioned in Table 1; in combination, to the best of our knowledge, Tables 1 and S1 comprise all published reports. For abbreviations see Table 1.

reference	zoogeographic realm	country	type	plant	insect	major news, quotes, comments
Hopkins (1927)	Oceanian	Samoa	I	BOR	DAN	p. 15: *Euploea schmeltzi* "is found in flocks of many hundreds on *Toumefortia argentea* L. [*H. foertherianum*]" "About 150 on one dead branch below the tree, all males." p. 16: "... many hundreds of *Euploea schmeltzi* on the fruit-clusters of *T. argentea* in Savai'i, but even in this case it was the dead and withered clusters that were preferred."
Poulton (1932)		Fiji				"... it is difficult to imagine in what the attraction lies; no exudation of any sort was observed."
Benoist (1933)	Neotropical	French Guyana			ARC	Found (in 1914) Syntomidae (ARC) at *Heliotropium* (as *Heliophytum*) and at uprooted plants which were identified with uncertainty (and likely erroneous) as *Cacoucia coccinfla* Aubl. (Combretaceae). [No details.]
Lever (1934)	Oceanian	Solomon Islands			DAN	"... Euploeas feeding on the recently dead, flaccid leaves of *Tournefortia*. Their proboscides were fully extended and they were actively engaged in feeding on the surface of the leaf." "... first definite observation of their actual feeding and the use of the proboscis".
Hoffmann (1936)	Neotropical	Brazil		AST	ARC	Mentions some ARC at "foul Seneciobushes" without any surprise. [No details.]
Poulton (1936)	Oceanian	Solomon Islands		BOR	DAN	*Euploea* taken on a broken-off branch of a *H. foertherianum* (as *T. argentea*).
Barnes (1939)	Oriental	India				Plants of *Cynoglossum denticulatum* "completely covered with *Danais melissa* "... engaged in vigorously scratching the surface of the leaves with the 'claws' of their front legs and drinking the sap thus made available ...".
Moss (1947)	Neotropical	Brazil	E		ARC DAN ITH	Tested, based on personal information by Hagmann (1938), *H. indicum* as bait and "found that the leaves, stalks, even the roots and especially the long seed racemes constitute a most remarkable attraction" "on one plant ... as many as 40 or 50 specimens". Some species appear only in one sex, male or female as the case may be; the opposite sex in a few kinds occasionally turning up as a rarity, in others, like *Aethria leucaspis*, one of the commonest, all males, never once a female." [See SI 7.]
Le Moult (1955)		French Guyana	I, E		ITH ARC	Incidentally noticed a plant, *Heliotropium* sp., with great numbers of "Synthomides" and "Ithomides", planted it in his garden and "was able to collect hundreds" specimens. [No details on species.]
Beebe & Kenedy (1957)		Trinidad	E		ARC	Nine species of ARC at "*Heliotropium indicum* Linnaeus is a common weed which, when uprooted and allowed to shrivel, exercises a remarkable selective attraction for several lepidopteran families including the Ctenuchidae. For some unknown reason, however, only certain species appear to be attracted to it, while other species common in the same locality ignore it completely." "*Pseudosphex kenedyae* [*Myrmecopsis*] is a common visitor to *H. indicum*, but it has not been seen elsewhere nor had we ever seen a specimen before using the attractant".
Fleming (1957)						*Pseudosphex kenedyae* sp. nov. [now *Myrmecopsis*] collected at *Heliotropium indicum*.
Fleming (1959)						*Aclytia leucaspila* sp. nov. and some other ARC collected at *Heliotropium*.
Gilbert & Ehrlich (1970)					DAN ITH	Baiting with *H. indicum*: "We were impressed that this plant attracted not only very large numbers of male ithomiines (*Ithomia drymo, Hymenitis andromica, Tithorea harmonia, Melinaea lilis, Hypothyris euclea, Mechanitis isthmia*, and others), but also large numbers of males of the danaine, *Lycorea ceres*." [No data.]
**Edgar *et al*. (1973), Edgar (1975), and Schneider *et al*. (1975)**: **recognition that attractiveness is due to the presence of 1,2-dehydropyrrolizidine ester alkaloids (PAs)**						
Masters (1971)	Neotropical	Venezuela	E	BOR	ITH ARC	Used *Heliotropium* to collect ITH and ARC; found 3 female *Heliconius cydno* (Helioconiinae) as a surprise. First and only observation of a *Heliconius* butterfly is dubious.
Owen (1971)	Afrotropical	Kenya	I		DAN	*Danaus chrysippus* at *Heliotropium* sp., previously damaged by browsing *Zonocerus* grasshoppers.
Edgar *et al*. (1973)	Australasian	Australia				*Danaus plexippus, D. hamatus* and *D. affinis* at *H. amplexicaule.* Mention that *Cynoglossum amabile* is also attrractive.

Adams & Goss (1975)	Nearctic	USA: Florida		ORC	ARC	*Epidendrum anceps* is exclusively pollinated by *Eucereon carolina*, *Lymire edwardsii* and *Cisseps fulvicollis* and *Oxydia vesulia* (Geometridae). [See SI 5.]
Amladi (1975)	Oriental	India		BOR	DAN	*Tirumala limniace* (as *Danaus*) and *Danaus chrysippus* at *H. indicum.* "At first I assumed that the butterflies were feeding on the flowers, but closer inspection revealed that all of them were clinging to, and feeding on, a dead and decaying inflorescence drooping from the plant." "I crushed an inflorescence bearing few flowers of this latter plant, sufficient to extract the plant juice without distorting its rigidity, and waited. In a few minutes all the 5 specimens of *D. limniace* transferred their attention to this plant and within seconds of arrival settled and avidly fed on the bruised portion of the plant." [Photograph.]
Schneider *et al*. (1975)	Afrotropical	Kenya	I, E	BOR	DAN	Many male *D. chrysippus* at withered *Heliotropium steudnerii.*
Goss & Adams (1976)	Nearctic	USA: Florida		ORC	ARC	Pollination experiments with *Epidendrum anceps.* [See SI 5.]
Bernays *et al*. (1977)	Afrotropical	Nigeria	I		DAN	Frequently witnessed *Danaus chrysippus* feeding on the juices of moribund *Zonocerus variegatus.*
Chathurvedi & Satheesan (1979)	Oriental	India		FAB	DAN	Many DAN (*Euploea core, Tirumala limniace* (as *Danaus*), *D genutia, D. chrysippus*) sucking withering leaves of *Crotalaria retusa,* also 4 males of *Hypolimnas bolia* which is a surprise and dubious.
Johnston & Johnston (1980)		Hong Kong				All local DAN found at *Crotalaria retusa.*
Lamas & Pérez (1981)	Neotropical	Peru	E		DAN ITH	25 of the 42 local DAN and ITH in Madre de Dios attracted to *Heliotropium indicum* baits. 94 % males.
Edgar (1982)	Oceanian	Solomon Island	I	APO BOR	DAN	*Species of Danaus and Euploea at Parsonsia spiralis and H. foertherianum.*
Boppré (1983)	Afrotropical	Kenya	I, E	BOR	DAN ARC	Scratching behaviour of *Danaus*, *Tirumala* and *Amauris* on living leaves of *H. pectinatum* that had been previously injured by leaf beetles. "Leaves scratched with fingernails or damaged by squeezing, instantly attracted Danainae and *Rhodogastria* [*Amerila*], respectively, but intact leaves offered simultaneously were always neglected."
**Boppré (1984): coining of the term "pharmacophagy"**						
Larsen (1986a)	Oceanian	Papua New Guinea	I	APO	DAN	Clusters of *Euploea* on dried vines of *Parsonsia lata*. Existing clusters appeared to act as visual cues for those that joined.
Larsen (1986b)				AST		*Danaus chrysippus* at flowers, cut stems and roots of *Ageratum conyzoides* were almost invariably males.
Boppré & Pitkin (1988)	Afrotropical	Kenya	E	pPA	DIP	Hundreds of Chloropidae of 4 genera (*Melanochaeta, Chlorops, Eutropha, Oscinella*) at dishes with pure PAs. [Photographs.]
Krasnoff & Dussourd (1989)	Nearctic	USA: Florida		pPA	ARC	Pure PAs, derivatives of PAs, and roots of *E. maculatum* attracted ARC.
Dussourd *et al*. (1989)				AST	DAN	Male *D. gilippus* are readily lureable with wilting *Eupatorium capillifolium* and consume offered crystalline monocrotaline *N*-oxide.
Boppré (1990)	Afrotropical	Kenya	I			"Flowers of the PA plant *Gynura scandens* are exclusively visited by PA-insects, and these exhibit the same sex bias as at withered plants or artificial baits; other Lepidoptera completely ignore these flowers."
DeVries & Stiles (1990)	Panamanian	Costa Rica	E	ORC	ITH ARC	Several ITH and ARC at flowers of *Epidendrum paniculatum.* [See SI 5.]
Davis & Barnes (1991)	Afrotropical	Mauritius	I	BOR	DAN	*Danaus chrysippus, Euploea euphon* and *Amauris phaedon* "imbibe exudations from the ends of broken branches" of *H. foertherianum* (as *Tournefortia argentea*).
Haribal (1992)	Oriental	India				DAN attracted to *Adelocaryum coelestinum* (as *Paracaryum*).
Chaturvedi (1994)						DAN attracted to *Trichodesma indicum* and *A. coelestinum* (as *Paracaryum*).
Calhoun (1996)	Nearctic	USA: Florida		—		*Danaus eresimus* and *D. gilippus* apparently gathering PAs around the cut stem of an unidentified plant.
Boppré & Fischer (1997)	Afrotropical	Ghana	E	AST BOR pPA	ORT	All stages of *Zonocerus variegatus* attracted to *Chromolaena odorata* and other PA-baits. [Little context on PAs.]
Häuser & Boppré (1997a)	Panamanian Neotropical	Costa Rica, Peru, Fr. Guyana		BOR pPA	ARC	More than 1,000 specimens of 98 species of ARC attracted to dry *Heliotropium* and dishes with pure PAs, extracted and purified from *Crotalaria scassellatii.* [List of species only for specimens from Costa Rica.]

Häuser & Boppré (1997b)						17 species of *Amerila* (including 5 sp. nov.) collected at PA-baits; recognition of a comb. nov. based on PA-baiting. [Little context on PAs.]
Scherer & Boppré (1997)	Afrotropical	Kenya		AST BOR pPA	COL	13 *Gabonia* sp. nov. and a *Nzerekorena* sp. nov. collected at *Heliotropium* bait, at *Ageratum, Gynura* and at pure PAs. [Little context on PAs.]
Jafer Palot *et al*. (1997)	Oriental	India	I	FAB BOR	DAN	Aggregating DAN on *C. peduncularis* and *H. indicum*. [Not seen, quoted by Nair (2003).]
Boppré (1999)	Afrotropical	Kenya	E		AGA	*Digama, Asota* (as *Aganais*) at various PA-baits. [Photographs of DAN, ARC, AGA at PA-sources. Little context on PAs.]
Grados (1999)	Neotropical	Peru		BOR	ARC	Survey with light and *Heliotropium* parallely. List of species provided but not differenciated.
Karthikeyan (1999)	Oriental	India	I	AST	DAN	Congreation of *E. core* on dry roots of *C. odorata.* "Is it possible that *Euploea core* acquire some alkaloids from the roots of *Chromolaena odorata* like they do by visiting *Heliotropium indicum* and other plants?" [No context.]
Conner *et al*. (2000)	Nearctic	USA: Florida	E		ARC	*Cosmosoma myrodora* collected at *E. capillifolium*.
Brevigonon (2003)	Neotropical	French Guyana	I	BOR	ITH	Ithomiines at roots of *Heliotropium* on a freshly ploughed field. [Brief mention of "alkaloids".]
Cock (2003)		Trinidad, Tabago	E		ARC	Mentions baiting with *Heliotropium* with reference to Beebe (1955) and provides colour photographs of *Dinia eagrus* and *Pseudsphex kenedyae* at bait. [No mention of PAs, no details or context.]
Nair (2003)	Oriental	India	I		DAN	Repeatedly observed *Euploea core* and *T. limniace* wlhich "seemed to rub their proboscids against the withered roots and appeared imbibing some substances, possibly alkaloids" of the roots of a fallen tree, determined as *Hopea parviflora* (Diptero-carpaceae). [First and only report re *Hopea*, which is not known to contain PAs; interesting report which requires verification.]
Freitas *et al.* (2003)	Neotropical	Brazil	E	BOR	DAN ITH ARC	Brief mention that *Heliotropium* plants can be used for baiting DAN, ITH, ARC. [Photographs of ITH at *Heliotropium.*]
Einem (2004)	Nearctic	USA: Texas	I		DAN	*Danaus gilippus* and *D. eresimus* at damaged *Heliotropium.* [Photographs.]
Grados (2004)	Neotropical	Peru	E		ARC	*Pitane* sp. nov. collected at *Heliotropium* baits. [No mention of PAs.]
Bhuyan *et al.* (2005)	Oriental	India		FAB	DAN	At *C. retusa*: "*D. genutia* spends more time on unopened flowers, by comparison to open flowers." [Study focusses on nectar host plant selection, no mention of PAs.]
Araújo (2006)	Neotropical	Brazil	E	BOR	ITH	Study on community of ITH mainly based on regular baiting with *Heliotropium*.
Mathew & Anto (2007)		India		FAB	DAN	Planted a garden "with butterfly aggregation plants such as *Heliotropium keralense* and *Crotalaria retusa* in order to facilitate roosting of danaid butterflies". "... the danaids formed large aggregations with populations ranging from 150 to 600 on *C. retusa* (Figure 5) and *H. keralense*". [PAs are mentioned but context incomplete.]
Prasad *et al.* (2008)	Oriental		I			Congregations (up to 60 specimens) of DAN (*Danaus, Euploea, Tirumala*) at *C. retusa*. "... the main purpose of aggregaton on *Crotalaria retusa* may be to imbibe the sap containing such [pyrrolizidine] alkaloids ...". [Context superficial.]
Honda (2008)		Japan		AST BOR	DAN	General discussion on DAN and PAs. Photographs of *Ideopsis similis* at *E. chinense*, *Parantica sita* at *H. foertherianum* (as *M. argentea*) and *E. mulciber* at an asteraceous plant.
Krauska (2009)	Nearctic	USA: Missouri		AST		Many *D. plexippus* at withering roots of *Gymnocoronis spilantoides.* [No mention of PAs.]
Das (2010)		India ?				Striking colour photographs of *Tirumala* at *Heliotropium.* [No mention of PAs.]
Mathew (2011)		India	I	BOR	DAN	Mentions adults of several DAN to visit *H. indicum*, *C. retusa*, *Trichodesma indicum* and *Adelocaryum coelestinum* (as *Paracaryum*) for pyrrolizidine alkaloids. [No context.]
ButterflyCircle (2012)	Oriental	Singapore		AST		Pictures of DAN at trimmings of *Artemisia scoparia*.
Rajesh *et al.* (2012)		India		FAB		Studied interactions of insects with *C. retusa*. "*D. chrysippus* and *E. core* visit the mature buds and flowers to collect nectar while *T. limniace* in huge aggregations collect sap from the stem and leaf petioles before flowering and after fruit set." "More than 800 individuals of this butterfly were found to aggregate on *C. retusa* plants for the collection of sap." Plants subsequently withered. [See Si 1.]

Lambkin (2013)	Oceanian	Papua New Guinea		BOR FAB		Three spp. of *Euploea* and *T. hamata* "in large numbers imbibing pyrrolizidine alkaloids from dead and damaged leaves and flowers of *Heliotropium foertherianum*". *Tirumala hamata* attracted to flowers and leaves of *C. retusa*. [Colour photographs; no context on PAs.]
Revathy & Mathew (2013)	Oriental	India				"Roosting of Danaid butterflies on *Crotalaria retusa* ... Aggregations of large populations of *Tirumala limniace*, *T. septentrionis*, *Parantica aglea*, and *Euploea core* etc., ...ranging from 250–500 on *C. retusa* and *H. keralense*." [Likely not roosting but gathering PAs; no mention of PAs.]
Chow (2014)		Singapore		AST FAB		*Danaus genutia* scratching on *C. retusa*, *Parantica* sp., *Ideopsis* sp., *Euploea* sp. at withered *A. scoparia*.<br>[Colour photographs; little context on PAs.]
Smith (2014)	Afrotropical	Kenya, Ghana	I, E	AST BOR FAB		(in 1975) "many hundreds of danaines ... all showing a fervid interest in *Crotalaria retusa*."<br>[Chapter on PAs and *Danaus* with several original observations; context, in part, too generalising; on p. 392 a table quoted as "from Gordon *et al.* 2010" which is not published in Gordon *et al.* (2010) but data is correct (Gordon, pers. comm.).]
Malcolm & Slager (2015)	Neotropical	Argentina	I	AST		"Thousands" of *D. erippus* nectaring at flowers of *Chromolaena arnottiana* in which PAs were found.
Mudai *et al.* (2015)	Oriental	India		BOR		"*Euploea doubledayi* ... visiting flowers of *Heliotropium indicum* even the dried plant of the species." [No mention of PAs.]
Hernández-Baz *et al.* (2016)	Neotropical	Colombia	E		ARC	*Trichura* spp. collected at *Heliotropium* sp. [No mention of PAs.]
Tan (2017)	Oriental	Singapore	I	FAB	DAN	*Danaus chrysippus* attracted to *H. indicum* and *C. retusa* L., at the latter they "claw the pea pod and then feeds on the fluids that the damaged pea pod exude". [No mention of PAs.]
Grados & Mantilla (2018)	Neotropical	Peru	E	BOR	ARC	Baited a *Theages* sp. with *Heliotropium* and mention several other ARC of which exclusively males were attracted. [No mention of PAs.]
Hawkeswood & Sommung (2018)	Oriental	Thailand	I	FAB	DAN	*Danaus chrysippus* at damaged stems and leaves of *Crotalaria spectabilis.* [Re PAs reference to Smith (2014) only.]
Boppré *et al*. (2020)	Neotropical	Peru	E	BOR	ARC	*Vanewrightia kiesela* gen. et sp. nov. collected at *Heliotropium* and other PA-baits. [Little context on PAs.]
Butterflies of Singapore (2020)	Oriental	Singapore	I	FAB	DAN	Colour photographs of DAN gathering PAs from Crotalaria retusa but other local species of *Crotalaria* appear not attractive. [PAs mentioned briefly.]
Grados *et al*. (2020)	Neotropical	Peru	E	BOR	ARC	*Corematura* spp. collected at *Heliotropium* sp. [No mention of PAs.]
Udaya *et al*. (2020)	Oriental	India		FAB	DAN	Planted *C. retusa* in a garden and observed congregations of *Tirumala*, *Parantica*, and *Euploea.* [Colour photographs; PAs mentioned briefly.]
Grados & Mantilla (2021)	Neotropical	Peru		BOR	ARC	*Centronia melanitis* collected at *Heliotropium* sp. [Context of PAs superficial.]
Lawson *et al*. (2021)	Nearctic	USA: Maryland	I	AST	DAN	*Danaus plexippus* at withered leaves of *E. serotinum* in a garden. [Review on *D. plexippus* and PAs.]
Grados & Ramírez (2021)	Neotropical	Peru	E	BOR	ARC	Several ARC baited with *Heliotropium*. [No context of PAs.]
Ancajima & Neyra-Hidalgo (2021)						Baited ARC with *Heliotropium* but with seemingly little success. [See SI 7 for comment.]
Badon (2021)	Oriental	Philippines	I		DAN	Pictures of *Euploea* at infructescences of *Heliotropium*. [Butterflies are not nectaring (as stated) but gathering PAs.]
Freitas *et al*. (2021)			E		DAN ITH	Practical tips for baiting DAN and ITH with *Heliotropium*. [Context incomplete.]
Silveira *et al*. (2023)	Neotropical	Brazil	I (E)	ORC	ITH ARC	Found *Epidendrum densiflorum* being pollinated exclusively by PA-pharmacophagous ITH and ARC. [See SI 5 for comment.]
Boppré (unpubl.)	Panamanian	Costa Rica	E	AST APO	DAN ARC	Roots of *Emilia* spp., *Erechtites hieracifolius, Prestonia* sp., and *Echites panduratus* are highly attractive.
Monzón & Boppré (unpubl.)	Neotropical	Peru				*Senecio burkartii* and *Prestonia* sp. are highly attractive.

Table S2

Insect taxa confirned to be attracted to pyrrolizidine alkaloids (PAs) as adults (see Tables 1 and S1) and their distribution. Comparing the distribution and the records clearly documents under-investigation which makes the list definitely provisional. Note that for the majority of the countries listed the currently available records are patchy: many represent incidental observations from a single habitat, often made on a single day, and mostly only a few specimens were recorded. See SI 7 for non-Lepidoptera that have hardly received attention.

**order**	**family**	subfamily	**tribe**	**sample taxa**	**distribution**	**records available from**
Lepidoptera	Nymphalidae	Danainae	Danaini	*Amauris*	Afrotropical	Kenya
				Danaus		Kenya, Ghana
					Neotropical, Panamanian	Argentina, Costa Rica, Peru
					Nearctic	Florida, Missouri, Texas
					Oceanian	Salomon Islands, Samoa, Tonga
					Oriental	India, Hong Kong, Papua New Guinea, Singapore, Sulawesi, Thailand
					Australasian	Australia
				Euploea	Oceanian	Fiji, Papua New Guinea, Solomon Islands, Samoa, Tonga
					Oriental	India, Philippines, Singapore, Sulawesi
					Australasian	Australia
				Ideopsis	Oriental	Malaysia, Sulawesi, Singapore, Japan
				Lycorea	Neotropical, Panamanian	Costa Rica, Ecuador, Guyana, Panama, Peru, Trinidad, Venezuela
				Parantica	Oriental	India, Singapore, Sulawesi
				Tirumala	Afrotropical	Kenya
					Oceanian	—
					Oriental	India, Singapore, Sulawesi
					Australasian	Australia
			Ithomiini	most genera	Neotropical, Panamanian	Brazil, Costa Rica, Ecuador, French Guyana, Panama, Peru, Trinidad, Venezuela
	Erebidae	Arctiinae	Amerilini	*Amerila*	Afrotropical	Kenya
					Oriental	Malaysia
					Australasian	—
			Arctiini	many Euchromiina	Nearctic	USA: Florida
					Afrotropical	Kenya, South Africa

					Oriental	Solomon Islands, Singapore
					Australasian	—
				many Ctenuchina	Neotropical, Panamanian	Argentina, Brazil, Columbia, Costa Rica, Ecuador, French Guyana, Panama, Peru, Trinidad, Venezuela
				some Phaegopterina		Argentina, Costa Rica, French Guyana, Peru, Trinidad
		Aganainae		*Asota, Digama*	Afrotropical	Kenya, South Africa
					Oriental	Malaysia, Singapore, Solomon Islands
					Australasian	—
Orthoptera	Pyrgomorphidae			*Zonocerus*	Afrotropical	Ghana, South Africa
Coleoptera	Chrysomelidae	Galerucinae	Alticini	*Gabonia, Nzerekorena*		Kenya
Diptera	Chloropidae			most spp. not yet indentified	worldwide	Costa Rica, Kenya, Peru, Venezuela

Table S3

Plants reported to be attractive for PA-phamacophagous insects (references in Tables 1,S1; Boppré *et al*. unpubl.) with vernascular names, global distribution, and references to selected chemical analyses. Due to under-investigation the Table is incomplete; other members of a given genus likely are attractive, too, and many more genera that produce 1,2-dehydropyrrolizidine ester alkaloids are known but observations related PA-pharmacophagy have yet not been made. PA-content often differs between organs. Additional vernascular names apply. The taxonomic status of many species has been in flux over the years and this will continue.

family	tribe / subfamily	genus / species	Fig. [1]	vernascular name(s)	distribution	sample references for chemistry
Apocynaceae	Apocyneae	*Amphineurion marginatum*			Oriental	Colegate *et al*. (2016)
	Echiteae	*Echites panduratus* (syn. *Fernaldia pandurata, Urechites karwinskii*)		loroco	Panamanian	Borges del Castilo *et al*. (1970), Colegate *et al.* (2016)
		Parsonsia spp. (*eucalyptophylla, lata, spiralis, straminea*)		silkpod	Oriental, Oceanian, Australian	Edgar & Culvenor (1975), Edgar *et al.* (1980)
		Prestonia spp. (*amabilis* and others)			Neotropical	Trigo & Brown (1990), Brehm *et al.* (2007)
	Nerieae	*Alafia* spp.			Afrotropical	Colegate *et al.* (2016)
Asteraceae	Anthemideae	*Artemisia* spp. (*capillaris*, *scoparia*)		false dill	temperate climates	Chen *et al.* (2019)
	Eupatorieae [2]	*Adenostemma* spp.			pantropical	Brown (1987)
		Ageratum conyzoides	3F	goat weed, tropical whiteweed	pantropical, partly invasive	Wiedenfeld & Röder (1991), Bosi *et al*. (2013), Wiedenfeld & Andrade-Cetto (2001)
		Aristeguietia gayana		asmachilca	parts of Neotropics	Colegate *et al*. (2015)
		Chromolaena odorata	3O	Siam weed	Panamanian, naturalised pantropical	Biller *et al*. (1994), Zachariades *et al*. (2009)
		Chromolaena (syn. *Eupatorium*) (*xestolepis, arnottiana*)			Americas	Malcolm & Slager (2015)
		Eupatorium [3] spp. (*capillifolium, chinense, coelestinum, maculatum, serotinum* and others)		boneset, mistflower, dogfennel	temperate regions of Northern hemisphere	Brown (1985), Colegate *et al*. (2018)
		Gymnocoronis spilanthoides [4]	3P,Q	Senegal tea plant, jazmín del bañado	Neotropical, naturalised in parts of USA, Australia, Asia, and Europe	Boppré & Colegate (2015)
		Neomirandea sp.			Neotropical	—
		Trichogoniopsis adenantha (as *Trichogonia gardeneri*)			Brazil	Brown (1984)
	Senecioneae [5]	*Emilia* spp. (*fosbergii, sonchifolia*)	3I,K	tasselflower, pualele	pantropical	Hsieh *et al.* (2015), Freitas *et al.* (2020)
		Erechtites hieraciifolius	3G,H	fireweed, American burnweed, pilewort	Americas	Manske (1939)
		Gynura scandens			Afrotropical	Wiedenfeld (1982)

		Senecio [6] spp. (*brasiliensis, burkartii*)		ragwort, groundsel	almost worldwide	Hirschmann *et al*. (1987), Klitzke & Trigo (2000), Sandini *et al*. (2013)
		Solanecio mannii (syn. *Crassocephalum*)			Afrotropical	Asres *et al*. (2008)
Boraginaceae	Boraginoideae [7]	*Cynoglossum* spp. (*amabile*, *denticulatum*)		hound's tongue	Old World, some in USA	El-Shazly *et al.* (1996)
		Myosotis spp.		forget-me-not	largely restricted to Western Eurasia, some spp. in Neotropics	Pedersen (1975), Resch *et al*. (1982)
		Adelocaryum (syn. *Paracaryum*) *coelestinum*			India	—
		Trichodesma indicum			pantropical	Ahmad *et al*. (2018)
	Heliotropoideae	*Heliotropium foertherianum* [8]	3A,B	velvetleaf soldier-bush, octopus bush, tree heliotrope	islands and coasts of Pacific and Indian ocean	Edgar (1982), Ogihara *et al*. (1997)
		Heliotropium spp. (*amplexicaule, angiospermum, curassavicum, indicum, keralense, pectinatum, steudnerii*)	3C,E	elephant tusk, crista de galo, fedegoso [9], rabo de alacrán, scorpion's tail	pantropical	Agnese *et al*. (1995), Souza *et al*. (2005)
		Tournefortia spp. (*gnaphalodes*)	3D	soldierbush	Neotropical	Brown (1985), Roque-Albelo *et al*. (2002)
Faba-ceae	Crotalarieae	*Crotalaria* spp. (*lanceolata, peduncularis, retusa, spectabilis, striata*)	3L–N	rattlebox	pantropical	Wiedenfeld *et al*. (1985), Williams & Molyneux (1987), Flores *et al*. (2009), Scupinari *et al*. (2020
Orchidaceae		*Epidendrum* [10] spp. (*anceps*, *densiflorum, paniculatum*)			Neotropical	—

[1] in main article; [2] Dominguez (1977); [3] The taxonomy of "*Eupatorium*" has often changed and is in flux; [4] USDA (2017); [5] Langel *et al*. (2011); [6] "*Senecio*" is one of the best investigated genus for PAs, taxonomy is in flux; [7] El-Shazly (2014); [8] see Manner & Elevich (2006); *Tournefortia argentifolia* is a Linnean herbarium name of 1777 which was never validly published; the respective plant is *Heliotropium foertherianum* Diane & Hilger 2003 which was originally described in 1782 as *Tournefortia argentea* and has homotypic synonyms (*Argusia argentea, Messerschmidia argentea*) and a recent heterotypic synonyme (*H. arboreum*, based on *Tournefortia arborea*); [9] name is also used for *Cassia occidentalis* (Leguminosae) which is not a PA-plant; [10] complex taxonomy in flux.

SI 9: References not contained in the main text

Abbott J (2014) Self-medication in insects: current evidence and future perspectives. Ecol Entomol 39:273–280

Abe F, Yamauchi T (1987) Parsonine, a pyrrolizidine alkaloid from *Parsonsia laevigata*. Chem Pharm Bull 35:4661–4663

Adams RM, Goss GJ (1975) The reproductive biology of epiphytic orchids of Florida III. *Epidendrum anceps* Jacquin. Am Orchid Soc Bull 45:488–492

Agnese M, Mellina S, Cabrera JL (1995) Pyrrolizidines alkaloids in medicinal plants sold in the city of Cordoba (Argentina): *Heliotropium curassavicum* L. Acta Farmaceutica Bonaerense 14:273–276 [in Spanish]

Ahmad L, He Y, Semotiuk AJ, Liu Q-R (2018) Qualitative determination of toxic pyrrolizidine alkaloids in *Trichodesma indicum*: A prevalent ethnomedicine of Northern Pakistan. J Compl Med Res 9:11–23

Aldrich JR, Chauhan K, Zhang Q-H (2016) Pharmacophagy in green lacewings (Neuroptera: Chrysopidae: *Chrysopa* spp.)? PeerJ 4:e1564

Almeida MR, Ravindran VK (1988) Relationship between the pyrrolizidine alkaloids, Danainae butterflies and *Ageratum conyzoides*. J Bombay Nat Hist Soc 85:241–242

Amano T, Nishida R, Kuwahara Y (1999) Pharmacophagous acquisition of clerodendrins by the turnip sawfly (*Athalia rosae ruficornis*) and their role in the mating behavior. Chemoecology 9:145–150

Amladi SR (1975) Danaid butterflies attracted to *Heliotropium indicum* (Boraginaceae), an alkaloid containing plant. J Bombay Nat Hist Soc 72:585–587

Ancajima GP, Neyra-Hidalgo DA (2021) Los Arctiini (Lepidoptera, Erebidae, Arctiinae) atraídos por *Heliotropium* sp. (Heliotropiaceae) en la Estación Biológica José Álvarez Alonso, Iquitos, Perú. Folia Amazónica 30:61–69

Asres K, Sporer F, Wink M (2008) Occurrence of pyrrolizidine alkaloids in three *Solanecio* species. Biochem Syst Ecol 36:399–407

Badon JAT (2021) Notes concerning the migration of *Euploea* (Lepidoptera: Nymphalidae: Danainae) on Palawan Island, Philippines. News Lep Soc 63:190–191, 208

Barnes E (1939) A curious habit of a danaid butterfly. J Bombay Nat Hist Soc 41:443

Beebe W, Kenedy R (1957) Habits, palatability and mimicry in thirteen ctenuchid moth species from Trinidad, B.W.I. Zoologica (New York) 42:147–158

Benoist R (1933) Deux plantes pièges pour les Lépidoptères en Guyane Francaise. Ann Soc entomol France 102:107–108

Bhuyan M, Kataki D, Deka M, Bhattacharyya PR (2005) Nectar host plant selection and floral probing by

the Indian butterfly *Danaus genutia* (Nympahlidae [sic; recte Nymphalidae]). J Res Lepid 38:79–84

Biller A, Boppré M, Witte L, Hartmann T (1994) Pyrrolizidine alkaloids in *Chromolaena odorata*. Chemical and chemoecological aspects. Phytochemistry 35:615–619

Bogner F, Boppré M (1989) Single cell recordings reveal hydroxydanaidal as the volatile compound attracting insects to pyrrolizidine alkaloids. Entomol exp & appl 50:171–184

Bogner F, Eisner T (1991) Chemical basis of egg cannibalism in a caterpillar (*Utetheisa ornatrix*). J Chem Ecol 17:2063–2075

Bogner F, Eisner T (1992) Chemical basis of pupal cannibalism in a caterpillar (*Utetheisa ornatrix*). Experientia 48:97–102

Boppré M (1983) Leaf-scratching – a specialized behaviour of danaine butterflies for gathering secondary plant substances. Oecologia 59:414–416

Boppré M (1995) Pharmakophagie: Drogen, Sex und Schmetterlinge. Biologie in unserer Zeit 25:8–17

Boppré M, Fischer OW (1997) *Zonocerus* and *Chromolaena* in West Africa. A chemoecological approach towards pest management. In: Krall S, Peveling R, Ba Diallo D (eds) New Strategies in Locust Control. Birkhäuser Verlag, CH-Basel, pp 265–279

Boppré M, Schneider D (1985) Pyrrolizidine alkaloids quantitatively regulate both scent organ morphogenesis and pheromone biosynthesis in male *Creatonotos* moths (Lep.: Arctiidae). J Comp Physiol 157:569–577

Boppré M, Vane-Wright RI, Wickler W (2017) A hypothesis to explain accuracy of wasp resemblance. Ecol Evol 7:73–81

Boppré M, Grados J, Laguerre M, Monzón J (2020) *Vanewrightia* gen. nov.—A highly variable taxon of Neotropical Ctenuchina (Lepidoptera: Erebidae: Arctiinae: Arctiini) revealed by behavioral traits. Insect Syst Divers 4(5):4; 1–19

Borges del Castilo J, Espana de Aguirre AG, Breton JL, Gonzalez AG, Trujilo J (1970) Loroquin, a new necine isolated from *Urechites karwinsky* Mueller (1-hydroxy-methylene-7-keto-dihydropyrrolizine). Tetrahedron Lett 11:1219–1220

Bosi CF, Rosa DW, Grougnet R, Lemonakis N, Halabalaki M, Skaltsounis AL, Biavatti MW (2013) Pyrrolizidine alkaloids in medicinal tea of *Ageratum conyzoides*. Rev Bras Farmac 23:425–432

Brevignon C (2003) Inventaire des Ithomiinae de Guyane Francaise (Lepidoptera, Nymphalidae). Lambillionea CIII:41–58

Brown KS jr (1987) Chemistry at the Solanaceae/Ithomiinae interface. Ann Missouri Bot Gard 74:359–397

Butterflies of Singapore (2020) Tigers, crows and the rattlebox plant. https://butterflycircle.blogspot.com/2020/10/tigers-crows-and-rattlebox-plant.html. Accessed 15 May 2023

ButterflyCircle (2012) Tigers-on-the-floor-on-the-trees-everywhere. http://www.butterflycircle.com/showthread.php?13463-Tigerson-the-floor-on-the-trees-everywhere. Accessed 15 May 2023

Calhoun JV (1996) Conquering soldiers: the successful invasion of Florida by *Danaus eresimus* (Lepidoptera: Nymphalidae). Holarctic Lepid 3:7–18

Chaturvedi N (1994) New adult male attractants of danaid butterflies. J Bomb Nat Hist Soc 91:466

Chaturvedi N, Satheesan SM (1979) Attraction of butterflies to *Crotalaria retusa* (Papilionaceae) at Khandala, Western Ghats. J Bomb Nat Hist Soc 76:534–535

Chen L-H, Wand JC, Guo Q-L, Qiao Y, Wand H-J, Liao Y-H, Sund D-A, Si J-Y (2019) Simultaneous determination and risk assessment of pyrrolizidine alkaloids in *Artemisia capillaris* Thunb. by UPLC-MS/MS together with chemometrics. Molecules 24:1077

Chow L (2014) Danainae butterflies – leaf scratching and withered plants. https://besgroup.org/2014/07/30/danainae-butterflies-leaf-scratching-and-withered-plants/. Accessed 15 May 2023

Colegate SM, Boppré M, Monzón J, Betz JM (2015) Pro-toxic pyrrolizidine alkaloids in the traditional Andean herbal medicine "asmachilca". J Ethnopharmacol 172:179–194

Colegate SM, Upton R, Gardner DR, Panter KE, Betz JM (2018) Potentially toxic pyrrolizidine alkaloids in *Eupatorium perfoliatum* and three related species. Implications for herbal use as boneset. Phytochem Anal 29(6):613–626

Conner WE, Jordan AT (2009) From armaments to ornaments: the relationship between chemical defense and sex in tiger moths. In: Conner WE (ed.) Tiger Moths and Woolly Bears. Behavior, Ecology, and Evolution of the Arctiidae. Oxford University Press, GB-Oxford, pp 155–172

Conner WE, Boada R, Schroeder FC, Gonzáles A, Meinwald J, Eisner T (2000) Chemical defense: bestowal of a nuptial alkaloidal garment by a male moth on its mate. Proc Natl Acad Sci USA 97:14406–14411

Culvenor CCJ, Edgar JA, Smith LW, Tweeddale HJ (1969) Dihydropyrrolizine analogues of pyrrolizidine alkaloids. Tetrathedron Lett 41:3599–3602

Culvenor CCJ, Edgar JA, Smith LW, Tweeddale HJ (1970) Dihydropyrrolizines. III. Preparation and reactions of derivatives related to pyrrolizidine alkaloids. Austr J Chem 23:1853–1867

Das K (2010) Butterfly migration. Slide 43. www.slideshare.net/kishendas/butterfly-migration-latestkishendas-4284839. Accessed May 15 2023

Dash GK, Abdullah MS (2013) A review on *Heliotropium indicum* L. (Boraginaceae). Intern J Pharm Sci Res 4:1253–1258

Davenport JW, Conner WE (2003) Dietary alkaloids and the development of androconial organs in *Estigmene acrea*. J Insect Sci 3:333

Davidson RB, Baker C, McElveen M, Conner WE (1997) Hydroxydanaidal and the courtship of *Haploa* (Lepidoptera: Arctiidae). J Lep Soc 51:288–294

Davis PMH, Barnes MJC (1991) The butterflies of Mauritius. J Res Lepid 30:145–161

de Jager ML, Anderson B (2019) When is resemblance mimicry? Funct Ecol 33:1586–1596

DeVries PJ, Stiles FG (1990) Attraction of pyrrolizidine alkaloid seeking Lepidoptera to *Epidendrum paniculatum* orchids and the potential effects on gene flow. Biotropica 22:290–297

Dobler S, Haberer W, Witte L, Hartmann T (2000) Selective sequestration of pyrrolizidine alkaloids from diverse host plants by *Longitarsus* flea beetles. J Chem Ecol 26:1281–1298

Dominguez XA (1977) Eupatorieae – chemical review. In: Heywood VH, Harborne JB, Turner BL (eds) The Biology and Chemistry of the Compositae. I. Academic Press, GB-London, pp 487–502

Downes JA (1973) Lepidoptera feeding at puddle-margins, dung, and carrion. J Lep Soc 27: 89–99

Edgar JA (1972) Dihydropyrrolizine secretions associated with coremata of *Utetheisa* moths (family Arctiidae). Experientia 38:627–628

Edgar JA, Culvenor CCJ (1975) Pyrrolizidine alkaloids in *Parsonsia* species (family Apocynaceae) which attract danaid butterflies. Experientia 31:393–394

Edgar JA, Eggers NJ, Jones AJ, Russell GB (1980) Unusual macrocyclic pyrrolizidine alkaloids from *Parsonsia heterophylla* A. Cunn and *Parsonsia spiralis* Wall. (Apocynaceae). Tetrahedron Lett 21:2657–2660

Einem GE (2004) Attraction of male queen butterflies to cardenolide- and alkaloid-containing plants during fall migrations. News Lep Soc 46:94–97

Eisner T (2005) For Love of Insects. Harvard University Press

El-Shazly A, Wink M (2014) Diversity of pyrrolizidine alkaloids in the Boraginaceae. Structures, distribution, and biological properties. Diversity 6:188–282

El-Shazly A, Sarg T, Ateya A, Aziz EA, Witte L, Wink M (1996) Pyrrolizidine alkaloids of *Cynoglossum officinale* and *Cynoglossum amabile* (family Boraginaceae). Biochem Syst Ecol 24:415–421

Extrasynthese (2023) Pyrrolizidine. https://www.extrasynthese.com/433-pyrrolizidine. Accessed 15 May 2023

Fleming H (1957) The Ctenuchidae (moths) of Trinidad, B.W.I. I. Euchromiinae. Zoologica 42:105–130

Fleming H (1959) The Ctenuchidae (moths) of Trinidad, B.W.I. Part II. Ctenuchinae. Zoologica 44:85–130

Flores AS, de Azevedo Tozzi AMG, Trigo JR (2009) Pyrrolizidine alkaloid profiles in *Crotalaria* species from Brazil: chemotaxonomic significance. Biochem Syst Ecol 37:459–469

Frei H, Lüthy J, Brauchli J, Zweifel U, Würgler FE, Schlatter C (1992) Structure/activity relationships of the genotoxic potencies of sixteen pyrrolizidine alkaloids assayed for the induction of somatic mutation and recombination in wing cells of *Drosophila melanogaster*. Chem-Biol Interactions 83:1–

22

Freitas AVL, Francini RB, Brown KS Jr (2003) Insetos como indicadores ambientais. In Cullen L Jr, Rudran R, Valladares-Pádua C (org.) Métodos de estudo em Biologia da Conservação e Manejo da Vida Silvestre. Fundação Boticário e Editora da UFPR, Curitiba, pp 125–151

Freitas JA, Ccana-Ccapatinta GV, Da Costa FB (2020) Pyrrolizidine alkaloids and other constituents from *Emilia fosbergii* Nicolson. Biochem Syst Ecol 92:104110

Freitas AVL, Santos JP, RRosa AHB, Iserhard CA, Richter A, Siewert RR, Gueratto PE, Carreira JYO, Lourenço GM (2021) Chapter 5. Sampling methods for butterflies (Lepidoptera). In: Santos JC, Fernandes GW (eds) Measuring Arthropod Diversity, Springer, pp 101–123

Fu PP, Yang YC, Xia Q, Chou MC, Cui YY, Lin G (2022) Pyrrolizidine alkaloids – tumorigenic components in Chinese herbal medicines and dietary supplements. J Food Drug Anal 10(4):198–211

Gilbert LE, Ehrlich, PR (1970) The affinities of the Ithomiinae and the Satyrinae (Nymphalidae). J Lep Soc 24:297–300

Goss GJ, Adams RM (1976) The reproductive biology of the epiphytic orchids of Florida IV. – Sexually selective attraction of moths to the floral fragrance of *Epidendrum anceps* Jacquin. Am Orchid Soc Bull 45*:*997–1001

Grados J (1999) Lista preliminar de los Ctenuchinae (Lepidoptera: Arctiidae) de la Zona Reservada Tambopata-Candamo, Madre de Dios, Perú. Rev per Entomol 41:9–14

Grados J (2004) Una nueva especvie de *Pitane* Walker, 1854 (Lepidoptera: Arctiidae) del sureste de Perú. Rev per Entomol 44:51–54

Grados J, Mantilla K (2018) Tres nuevas especies del género *Theages* Walker, 1855 (Lepidoptera: Erebidae: Arctiini) de Perú y Ecuador. Rev per biol 25:11–22

Grados J, Mantilla K (2021) Redescription of *Centronia melanitis* Hübner, 1818 (Lepidoptera: Arctiinae: Arctiini: Ctenuchina) with notes on androconial organs. Entomol Comm 3:ec03029

Grados J, Ramírez JJ (2021) Lista anotada de los Euchromiina "polillas avispas" (Lepidoptera: Erebidae: Arctiinae: Arctiini) del departamento de Loreto (Perú), con el reporte de nuevos registros y sinónimos. Rev per biol 38:e21906

Grados J, Ramírez JJ, Farfán J, Cerdeña J (2020) Contribution to the knowledge of the genus *Corematura* Butler, 1876 in Peru, with the report of a new synonym (Lepidoptera: Erebidae, Arctiinae, Arctiini, Ctenuchina). SHILAP Rev Lepid 48:71–82

Guilford T (1994) "Go-slow" signalling and the problem of automimicry. J Theoret Biol 170*:*311–316

Haribal M (1992) The Butterflies of Sikkim Himalaya and Their Natural History. Sikkim Nature Conservation Foundation (SNCF), Gangtok, Sikkim. 217 pp + 60 pl. [Not seen, quoted by Nair (2003)]

Hawkeswood TJ, Sommung B (2018) Observations on feeding by adults of the plain tiger, *Danaus*

chrysippus chrysippus (L, 1758) (Lepidoptera: Nymphalidae) in Bangkok, Thailand, with a review of some literature dealing with flower visitation by the species. Calodema 602:1–5

Hirschmann GS, Ferro EA, Franco L, Recalde L, Theoduloz C (1987) Pyrrolizidine alkaloids *from Senecio brasiliensis* populations. J Nat Prod 50:770–772

Hoffmann F (1936) Beiträge zur Lepidopterenfauna von Sta. Catharina. Syntomidae. Entomol Rundsch 53 (30, 31):435–439, 446–452

Hopkins GHE (1927) *Euploea schmeltzi schmeltzi*. In: Insects of Samoa and other Samoan terrestrial Arthropoda. Part III, Lepidoptera, Fasc. 1. The British Museum (Natural History), GB-London

Hsieh C-H, Chen H-W, Lee C-C, He B-J, Yang Y-C (2015) Hepatotoxic pyrrolizidine alkaloids in *Emilia sonchifolia* from Taiwan. J Food Comp Anal 42:1–7

Jafer Palot M, Mathew G, Zacharias VJ (1997) Butterflies of Periyar Tiger Reserve, Kerala (India). In: Prakash R (ed.) Advances in Forestry Research in India. International Book Distributors, Dehra Dun, pp 188–204 [Not seen, quoted by Nair 2003]

Johnson G, Johnson B (1980) This is Hong Kong. Butterflies. Crown Copyright Reserved Hong Kong

Jordan AT, Conner WE (2007) Dietary basis for developmental plasticity of an androconial structure in the salt marsh moth *Estigmene acrea* (Drury) (Lepidoptera: Arctiidae). J Lep Soc 61:32–37

Karthikeyan S (1999) Congregation of common crow *Euploea core* butterflies at Bannerghatta National Park. J Bombay Nat Hist Soc 96:486

Klitzke CF, Trigo JR (2000) New records of pyrrolizidine alkaloid-feeding insects. Hemiptera and Coleoptera on *Senecio brasiliensis*. Biochem Syst Ecol 28:313–328

Kopp T, Abdel-Tawab M, Mizaikoff B (2020) Extracting and analyzing pyrrolizidine alkaloids in medicinal plants: a review. Toxins 12:320

Krasnoff SB, Roelofs WL (1989) Quantitative and qualitative effects of larval diet on the male scent secretions of *Estigmene acrea*, *Phragmatobia fuliginosa* and *Pyrrharctia isabella* (Lepidoptera: Arctiidae). J Chem Ecol 15:1077–1093

Lambkin TA (2013) A revision of the *Euploea batesii* C. and R. Felder, 1865 'complex' (Lepidoptera: Nymphalidae: Danainae) in mainland Papua New Guinea and Australia, including its biology and biogeography. Austr Entomol 40:187–218

Langel D, Ober D, Pelser PB (2011) The evolution of pyrrolizidine alkaloid biosynthesis and diversity in the Senecioneae. Phytochem Rev 10:3–74

Larsen TB (1986b) *Ageratum conyzoides* (Compositae) indirectly confirmed as a source of pyrrolizidine alkaloids. J Bombay Nat Hist Soc 96:458–459

Latoxan (2023) https://www.latoxan.com/moleculars_product.php?id=673&n=6. Accessed 15 May 2023

Le Moult E (1955) Mes chasses aux papillons. Éditions Pierre Horay, F-Paris

Lever RA (1934) On a euploeine association on a small island near Santa Isabel in the Solomons; also a

further observation on Euploeas attracted by dead leaves of *Tournefortia*. Proc R entomol Soc Lond 9:57

Lindigkeit R, Biller A, Buch M, Schiebel H-M, Boppré M, Hartmann T (1997) The two faces of pyrrolizidine alkaloids: a tertiary amine and its *N*-oxide and their role in chemical defense of insects with acquired plant alkaloids. Europ J Biochem 245:626–636

Loaiza JCM, Céspedes CL, Beuerle T, Theuring C, Hartmann T (2007) *Ceroplastes albolineatus*, the first scale insect shown to sequester pyrrolizidine alkaloids from its host-plant *Pittocaulon praecox*. Chemoecology 17:109–115

Macel M, Klinkhamer PG, Vrieling K, van der Meijden E (2002) Diversity of pyrrolizidine alkaloids in *Senecio* species does not affect the specialist herbivore *Tyria jacobaeae*. Oecologia 133:541–550

Malcolm SM, Slager BH (2015) Migration and host plant use by the Southern monarch, *Danaus erippus*. In: Oberhauser KS, Neil KR, Altizer S (eds) Monarchs in a Changing World. Biology and Conservation of an Iconic Butterfly, Cornell University Press, Ithaca & London, pp 225–235

Manner HI, Elevitch CR (2006) *Tournefortia argentea* (tree heliotrope). In: Elevitch CR (ed.) Traditional Trees of Pacific Islands: Their Culture, Environment, and Use. https://agroforestry.org/images/pdfs/Tournefortia-treeheliotr.pdf. Accessed 15 May 2023

Manske RHF (1939) The alkaloids of *Senecio* species. IV. *Erechtites hieracifolia* (L.) Raf. Can J Res 17B:8–9

Martins CHZ, Trigo JR (2016) Pyrrolizidine alkaloids in the pericopine moth *Scearctia figulina* (Erebidae: Arctiinae): metabolism and chemical defense. J Bras Chem Soc 27:1437–1443

Masters JH (1971) *Heliconius cydno* (Lepidoptera: Nymphalidae) attracted to heliotrope. Entomol News 82:135–136

Masters AR (1991) Dual role of pyrrolizidine alkaloids in nectar. J Chem Ecol 17:195–205

Mathew G (2011) A Handbook on the Butterflies of Nilgiri Biosphere reserve. KFRI Research Report No. 398, Kerala Research Institute, India

Mathew G, Anto M (2007) In situ conservation of butterflies through establishment of butterfly gardens: A case study at Peechi, Kerala, India. Current Sci 93:337–347

Molleman F (2010) Puddling: from natural history to understanding how it affects fitness. Entomol exp & appl 134:107–113

Mudai P, Kalita J, Das GN, Boruah B (2015) Notes on some interesting butterflies (Lepidoptera) from Nambor-Doigrung wildlife Sanctuary, North East India. J Entomol Zool Studies 3:455–468

Nair VP (2003) Congregations of common crow butterflies *Euploea core* Cramer at Aralam Wildlife Sanctuary, Kerala. J Bombay Nat Hist Soc 100:632

Narberhaus I, Zintgraf V, Dobler S (2005) Pyrrolizidine alkaloids on three trophic levels - evidence for toxic and deterrent effects of phytophages and predators. Chemoecology 15:121–125

Neuman MG, Cohen LB, Opris M, Nanau R, Jeong H (2015) Hepatotoxicity of pyrrolizidine alkaloids. J Pharm Pharm Sci 18:825–843

Nishida R, Fukami H (1990) Sequestration of distasteful compounds by some pharmacophagous insects. J Chem Ecol 16:151–165

Nishida R, Kawai K, Amano T, Kuwahara Y (2004) Pharmacophagous feeding stimulant activity of *neo*-cleodane diterpenoids for the turnip sawfly, *Athalia rosae ruficornis*. Biochem Syst Ecol 32:15–25

Nuringtyas TR, Verpoorte R, Klinkhamer PGL, van Oers MM, Leiss KA (2014) Toxicity of pyrrolizidine alkaloids to *Spodoptera exigua* using insect cell lines and injection bioassays. J Chem Ecol 40:609–616

Ogihara K, Miyagi Y, Higa M, Yogi S (1997) Pyrrolizidine alkaloids from *Messerschmidia argentea*. Phytochemistry 44:545–547

Opitz SEW, Müller C (2009) Plant chemistry and insect sequestration. Chemoecology 19:117–154

Owen DF (1971) Tropical Butterflies. Clarendon Press, Oxford

Pansarin ER (2003) Biologia reprodutiva e polinização em *Epidendrum paniculatum* Ruiz & Pavón (Orchidaceae). Rev Bras Bot 26:203–211

Pasteels JM, Theuring C, Witte L, Hartmann T (2003) Sequestration and metabolism of protoxic pyrrolizidine alkaloids by larvae of the leaf beetle *Platyphora boucardi* and their transfer via pupae into defensive secretion of adults. J Chem Ecol 269:337–355

Pedersen E (1975) Pyrrolizidine alkaloids in Danish species of the family Boraginaceae. Arch Pharm Chem Sci Ed 3:55–64

Plotkin D, Goddard J (2013) Blood, sweat, and tears: a review of the hematophagous, sudophagous, and lachryphagous Lepidoptera. J Vector Ecol 38:289–294

Poulton EB (1932) Observations by R.A. Lever and H.W. Simmonds on male euploeine butterflies attracted to the "forget-me-not tree," *Tournefortia argentea,* L., in Fiji. Proc Entomol Soc Lond 6:77–80

Poulton EB (1936) Euploeine butterflies feeding at the broken surface of a *Tournefortia* branch, and some days later on withered leaves of the same branch: Tulagi, Solomon Islands, 1936, R.A. Lever. Proc R Entomol Soc (A) 11:94–95

Prasad VR, Kothari MJ, Barne KB, Rao PSN (2008) Congregation of danaid butterflies on *Crotalaria retusa* L. (Fabaceae). Nelumbo (Bull Bot Survey India) 50:193–195

Rajesh B, Sunanda Devi D, Sandhya Rani D, Zafar R, Govinda Rao N, Survarna Raju P, Solomon Raju AJ, Ravikumar G (2012) A study on interactions of insects with *Crotalaria retusa* L. (Fabaceae). Adv Pollen Spore Res 30:103–110

Ramos BCM, Trigo JR, Rodrigues D (2019) The specialization continuum: decision-making in butterflies with different diet requirements. Behav Proc 165:14–22

Ramos BCM, Trigo JR, Rodrigues D (2020) *Danaus* butterflies of the Americas do not perform leaf-scratching. Arthropod-Plant Interactions 14:521–529

Reina M, Mericli AH, Gonzáles-Coloma A (1998) A minor pyrrolizidine alkaloid from *Heliotropium bovei*. Nat Prod Lett 11:291–296

Resch JF, Rosenberger DF, Meinwald J (1982) Biologically active pyrrolizidine alkaloids from the true forget-me-not, *Myosotis scorpioides*. J Nat Prod 45:358–362

Revathy VS, Mathew G (2013) Seasonality of Rhopalocera (Lepidoptera) species in the butterfly garden at Nilambur in Kerala, southern India. Colemania 35:1–9

Roeder E (1995) Medicinal plants in Europe containing pyrrolizidine alkaloids. Pharmazie 50:83–98

Roeder E (2000) Medicinal plants in China containing pyrrolizidine alkaloids. Pharmazie 55:711–726

Roeder E, Wiedenfeld H (2011) Pyrrolizidine alkaloids in plants used in the traditional medicine of Madagascar and the Mascarene islands. Pharmazie 66:637–647

Roeder E, Wiedenfeld H (2013) Plants containing pyrrolizidine alkaloids used in the traditional Indian medicine—Including ayurveda. Pharmazie 68:83–92

Roque-Albelo L, Schroeder FC, Conner WE, Bezzerides A, Hoebeke ER, Meinwald J, Eisner T (2002) Chemical defense and aposematism: the case of *Utetheisa galapagensis*. Chemoecology 12:153–157

Rothschild M, Aplin RT, Cockrum PA, Edgar JA, Fairweather P, Lees R (1979) Pyrrolizidine alkaloids in arctiid moths (Lep.) with a discussion on host plant relationships and the role of these secondary plant substances in the Arctiidae. Biol J Linn Soc 12:305–326

Sandini TM, Berto MSU, de Souza Spinosa H (2013) *Senecio brasiliensis* e alcaloides pirrolizidínicos: toxicidade em animais e na saúde humana. Biotemas 26:83–92

Santos TMR, Shapiro JT, Shibuya PS, Aoki C (2013) Observation of *Trigona recursa* Smith (Hymenoptera: Apidae) feeding on *Crotalaria micans* Link (Fabaceae: Faboideae) in a Brazilian savanna fragment. Sociobiology 60:210–213

Schneider D, Boppré M, Zweig J, Horsley SB, Bell TW, Meinwald J, Hansen K, Diehl EW (1982) Scent organ development in *Creatonotos* moths: regulation by pyrrolizidine alkaloids. Science 215:1254–1255

Schramm S, Köhler N, Rozhon W (2019) Pyrrolizidine akaloids: biosynthesis, biological activities and occurrence in crop plants. Molecules 24:498

Sehlmeyer S, Wang L, Langel D, Heckel DG, Mohagheghi H, Petschenka G, Ober D (2010) Flavin-dependent monooxygenases as a detoxification mechanism in insects: new insights from the arctiids (Lepidoptera). PLoS One 5:e10435

Selleck (2023) https://www.selleckchem.com/products/monocrotaline.html. Accessed 15 May 2023

SIGMA-ALDRICH (2023) https://www.sigmaaldrich.com/DE/de/product/sial/phl80403. Accessed 15 May 2023

Silveira RS, Singer RB, Ferro VG (2023) Pollination in *Epidendrum densiflorum* Hook. (Orchidaceae: Laeliinae): fraudulent trap-flowers, self-incompatibility, and a possible new type of mimicry. Plants 12:679

Simmons R (2009) Adaptive coloration and mimicry. In: Conner WE (ed.) Tiger Moths and Woolly Bears. Behavior, Ecology, and Evolution of the Arctiidae. Oxford University Press, GB-Oxford, pp 115–126

Simmons RB, Weller S (2002) What kinds of signals do mimetic tiger moths send? A phylogenetic test of wasp mimicry. Proc R Soc B269:983–990

Singh P, Grone N, Tewes LJ, Müller C (2022) Chemical defense acquired via pharmacophagy can lead to protection from predation for conspecifics in a sawfly. Proc R Soc B289:20220176

Smith DAS (2014) African Queens and Their Kin: a Forty-Year Darwinian Odyssey. Brambleby Books

Souza JSN, Machado LL, Pessoa ODL, Braz-Filho R, Overk CR, Yao P, Cordell GA, Lemos TLG (2005) Pyrrolizidine alkaloids from *Heliotropium indicum*. J Braz Chem Soc 16:1410–1415

Speed MP, Ruxton GD, Mappes J, Sherratt TN (2012) Why are defensive toxins so variable? An evolutionary perspective. Biol Rev 87:874–884

Stamm P, Mann F, McClure M, Elias M, Schulz S (2019) Chemistry of the androconial secretion of the ithomiine butterfly *Oleria onega*. J Chem Ecol 45:768–778

Svennungsen TO, Holen ØH (2007) The evolutionary stability of automimicry. Proc R Soc B274:2055–2065

Tan H (2017) Butterflies of Singapore. *Danaus chrysippus*. http://butterflycircle.blogspot.com.au/2016/10/butterfly-of-month-october-2016.html Accessed 15 May 2023

Thallamy DW, Mullin CA, Frazier JL (1999) An alternate route to insect pharmacophagy: the loose receptor hypothesis. J Chem Ecol 25:1987–1997

Trigo JR, Brown KS jr (1990) Variation of pyrrolizidine alkaloids in Ithomiinae: a comparative study between species feeding on Apocynaceae and Solanaceae. Chemoecology 1:22–29

Trigo JR, Witte L, Brown KS jr, Hartmann T, Barata LES (1993) Pyrrolizidine alkaloids in the arctiid moth *Hyalurga syma*. J Chem Ecol 19:669–679

Trigo JR, Brown KS jr, Henriques SA, Barata LES (1996) Quantitative patterns of pyrrolizidine alkaloids in Ithomiinae butterflies. Biochem Syst Ecol 24:181–188

Turner JRG (1984) Mimicry: the palatability spectrum and its consequences. In: Vane-Wright RI, Ackery PR (eds) The Biology of Butterflies. Academic Press, GB-London, pp 141–161. = Symp R Entomol Soc 11; reprinted 1989 by Princeton University Press

Udaya KK, Bharath S, Nagaraj SS, Sankar TMV (2020) Congregation of danaine butterflies (Nymphalidae) on *Crotalaria retusa* following post monsoon migration. My Forest (Karnataka

Forest Department Bengaluru) 56:39–44

Wei X, Vrieling K, Mulder PPJ, Klinkhamer PGL (2015) Testing the generalist-specialist dilemma: the role of pyrrolizidine alkaloids in resistance to invertebrate herbivores in *Jacobaea* species. J Chem Ecol 41:149–167

Weller SJ, Simmons RB, Boada R, Conner WE (2000) Abdominal modifications occurring in wasp mimics of the Ctenuchine-Euchromiine clade (Lepidoptera: Arctiidae). Ann Entomol Soc Am 93:920–924

Wiedenfeld H (1982) Two pyrrolizidine alkaloids from *Gynura scandens*. Phytochemistry 21:2767–2768

Wiedenfeld H, Andrade-Cetto A (2001) Pyrrolizidine alkaloids from *Ageratum houstonianum* Mill. Phytochemistry 57:1269–1271

Wiedenfeld H, Röder H (1991) Pyrrolizidine alkaloids from *Ageratum conyzoides*. Planta Medica 57:578–579

Wiedenfeld H, Röder E, Anders E (1985) Pyrrolizidine alkaloids from seeds of *Crotalaria scassellatii*. Phytochemistry 24:376–378

Williams MC, Molyneux RJ (1987) Occurrence, concentration, and toxicity of pyrrolizidine alkaloids in *Crotalaria* seeds. Weed Sci 35:476–481

Witte L, Ehmke A, Hartmann T (1990) Interspecific flow of pyrrolizidine alkaloids. Naturwissenschaften 77:540–543
